# High Fungal Diversity but Low Seasonal Dynamics and Ectomycorrhizal Abundance in a Mountain Beech Forest

**DOI:** 10.1007/s00248-021-01736-5

**Published:** 2021-03-23

**Authors:** Markus Gorfer, Mathias Mayer, Harald Berger, Boris Rewald, Claudia Tallian, Bradley Matthews, Hans Sandén, Klaus Katzensteiner, Douglas L. Godbold

**Affiliations:** 1 Bioresources Unit, Center for Health & Bioresources, AIT Austrian Institute of Technology GmbH, Vienna, Austria; 2 Forest Ecology, Dept. of Forest and Soil Sciences, University of Natural Resources and Life Sciences (BOKU), Peter-Jordan-Strasse 82, 1190 Vienna, Austria; 3 Forest Soils and Biogeochemistry, Swiss Federal Institute for Forest, Snow and Landscape Research (WSL), Birmensdorf, Switzerland; 4 Symbiocyte, Vienna, Austria; 5 Environment Agency Austria, Vienna, Austria

**Keywords:** Ectomycorrhiza, Mountainous beech forest, Plant pathogenic fungi, Saprotrophic fungi, Soil fungal communities, Spatial and seasonal diversity

## Abstract

Forests on steep slopes constitute a significant proportion of European mountain areas and are important as production and protection forests. This study describes the soil fungal community structure in a European beech-dominated mountain forest stands in the Northern Calcareous Alps and investigates how it is determined by season and soil properties. Samples were collected at high spatial resolution in an area of ca. 100 m × 700 m in May (spring) and August (summer). Illumina MiSeq high-throughput sequencing of the ITS2-region revealed distinct patterns for the soil fungal communities. In contrast to other studies from temperate European beech forest stands, Ascomycota dominated the highly diverse fungal community, while ectomycorrhizal fungi were of lower abundance. Russulaceae, which are often among the dominant ectomycorrhizal fungi associated with European beech, were absent from all samples. Potentially plant pathogenic fungi were more prevalent than previously reported. Only subtle seasonal differences were found between fungal communities in spring and summer. Especially, dominant saprotrophic taxa were largely unaffected by season, while slightly stronger effects were observed for ectomycorrhizal fungi. Soil characteristics like pH and organic carbon content, on the other hand, strongly shaped abundant taxa among the saprotrophic fungal community.

**Supplementary Information:**

The online version contains supplementary material available at 10.1007/s00248-021-01736-5.

## Introduction

Approximately 40% of European mountain areas are covered by forests [1]. In comparison to low-elevation forests, mountain forests are generally exposed to more extreme environmental conditions, such as higher solar radiation and precipitation, lower annual temperatures, harsher winters, and avalanches [2]. Although European mountain forest ecosystems are in a relatively natural state, they currently undergo major compositional and structural shifts—partly as a result of changes in land use, climate, and natural forest disturbance regimes [3]. European beech is the most abundant broad-leaved tree species in Central Europe [4] and is a major constituent of Alpine forests. A third of the current potential beech forest area in Europe is located on calcareous soils and considered especially vulnerable to future climate scenarios [5, 6]. Mountain forest soils developed on calcareous substrates, in particular from limestone and dolomite, are usually shallow, have high organic carbon and Ca contents, and near-neutral pH values [7, 8]. While there is an increasing number of studies on soil processes in spruce or mixed mountain forests (on calcareous bedrock) [9, 10], the knowledge base on shallow, calcareous beech forest soils in mountainous areas is still limited.

The steep slopes and varying topographies of mountain forests result in heterogeneity in microsite and microclimatic conditions, as well as interlocking patterns of different humus and soil types [11]. This causes also heterogeneity in ecosystem processes such as N mineralization, nitrification, and consequently N availability [12]. High heterogeneity in site properties and stand characteristics provides a variety of ecological niches and promotes a high (aboveground) biodiversity [13, 14]. Calcareous forests in the European Alps are hotspots for vascular plant diversity [15]. In contrast to vascular plant diversity, which has been studied across all European forest types [15], studies of soil microbial diversity have been carried out mainly on homogeneous low-elevation forests [16–18] (but see [10]). These studies have primarily considered fertility gradients or the effects of understory vegetation [16–18]. Although the importance of spatial heterogeneity for microbial community composition has been widely recognized [19], it is not fully understood whether observations at large scales apply to stand-levels [20], particularly for calcareous mountain forests with high soil spatial heterogeneity [21].

Soil fungi are a key component of microbial communities in forest ecosystems, especially as integral constituents of carbon (C) and nutrient cycling [22, 23]. DNA sequence-based methods triggered manifold developments in fungal taxonomy [e.g., 24] and enabled recent surveys of soil fungi from a range of forests—revealing an extremely high soil fungal diversity [e.g., 25, 26]. Three major functional groups (“guilds”) of soil fungi can be distinguished according to their ecological lifestyle [27], i.e., saprotrophic, symbiotic, and plant pathogenic fungi. Symbiotic ectomycorrhizal (ECM) fungi acquire carbohydrates from living host plants [28] and often have a strong ecosystem- and host-specificity [25, 26]. Their abundance, diversity, and activity can be strongly related to seasonal changes in photosynthetic activity in accordance with the phenological stages of the host plant [29–32]. Soil calcium concentration has been reported to influence the taxonomic richness of ECM symbionts on *Alnus* spp. [33]. Saprotrophic fungi rely for their nutrition entirely on dead organic matter and are therefore especially sensitive to quality and quantity of organic matter [34, 35], but see [36] but were also reported to be affected by potential evapotranspiration [37]. In saprotrophic fungi, mycelia growth peaked in autumn and winter [10]. Plant pathogenic fungi in soil make up the bulk of microbes attacking roots [38] and are dominantly non-host-specific necrotrophs, i.e., killing host tissue and thereby providing conditions favorable to their growth and sporulation. Štursová et al. recently found no indication of seasonal patterns of plant pathogenic fungal activity below ground in a mountainous, *Picea abies* forest [10].

Relative to the functions that soil fungi perform, there is a very limited understanding of the spatial and temporal variations of soil fungal communities and their driving forces [32]. Climatic conditions, plant species composition, and soil factors have been identified as key parameters shaping the functional and taxonomic composition of soil fungal communities. The main soil factors include soil C, nutrient availability, and pH [e.g., 25, 26, 37, 39, 40]. This study describes the soil fungal community structure in a mountain beech forest and investigates how it is determined by plant phenological changes concomitant with season and soil properties. It is hypothesized that (1) soil conditions on calcareous bedrock in conjunction with a highly heterogeneous topology of mountain forests harbor a distinct and diverse fungal community; during the short growing season in mountain beech forests, the compositions of (2) symbiotic and plant pathogenic fungal communities are subject to pronounced seasonal changes, while (3) saprotrophic fungal community composition is primarily determined by soil properties.

## Materials and Methods

### Study Site and Soil Sampling

The “Molln” experimental site is located in the Reichraminger Hintergebirge, a mountain range located in the Northern Calcareous Alps of Austria (47° 49′ 08″ N, 14° 23′ 34″ E). The steeply sloping site (35°) is south exposed, ranging from 1000 to 1100 m a.s.l.; average annual air temperature and precipitation are 7.8°C and 1645 mm. The site is dominated by European beech (*Fagus sylvatica* L.) and sparsely intermixed by Norway spruce (*Picea abies* (L.) H. Karst), sycamore (*Acer pseudoplatanus* L.), European ash (*Fraxinus excelsior* L.), and silver fir (*Abies alba* Mill.). Stand age was 146 years in 2015 [41]. Sparse layers of understory tree seedlings, grasses, and herbs were present. The calcareous parent bedrock is limestone and the dominant soil types are Rendzic Leptosol and Chromic Cambisol [42]. Soils are shallow (mean ~30 cm, max. ~50 cm) with a high rock content; the predominant humus form is Moder. See Online Resource 1 (Supplementary Materials and Methods, Figure S1, and Table S1) for details on (understory) vegetation, microclimate (i.e., temperature and precipitation from January to August 2015), and soil parameters.

Soil sampling took place during two sampling campaigns in May and August 2015, representing two key phenological growth stages of beech trees in the studied mountainous ecosystem coming along with seasonal changes. In May (i.e., “spring”), ~20% of leaves were unfolded (but not yet at full size) on most trees (BBCH12); in August (“late summer”), the foliage was still green and terminal buds were developed (BBCH91) before signs of senescence started occurring in September (i.e., beginning of leaf discoloration and isolated leaf fall) [43]. In late April 2015, 16 plots (~ 25 m × 25 m) were established at the experimental site; at each plot, four subplots were selected by choosing triangular areas between three mature beech trees (Online Resource 1, Figure S2). In May, three out of four subplots per plot were sampled, resulting in 48 samples. In August, all 64 subplots were sampled. A distance of ≥ 1.5 m was kept to beech trees; no other tree species were in close vicinity to sampling points. For soil sampling, we focused on the mineral topsoil (A-horizon), as the organic horizons LF (litter/fragmented litter) and H (humus) were often rather shallow and highly heterogeneous in extend (data not shown), and the mineral topsoil layer featured the highest carbon accumulation compared to deeper mineral soil layers (Online Resource 1, Table S1). After the organic horizons were removed carefully, approximately 1 l of soil was collected from the upper 10 cm of the mineral soil within each subplot. To mark sampled locations, pits were backfilled with quartz sand. Sampling tools were wiped clean to minimize cross-contamination between (sub-)plots. Soil samples were sieved (2 mm) and homogenized in the field; sieves were thoroughly rinsed in tap water and air-dried between sample processing. For microbial community analysis, 0.5 g fresh soil was weighed immediately into 1.5 ml LifeGuard Soil Preservation Solution (MO BIO, Carlsbad, CA, USA) on site. Soil samples for chemical analyses were stored fresh in sealed plastic bags. All samples were kept at 4°C until further processing.

### Soil Parameter and Molecular Fungal Community Analysis

In the laboratory, gravimetric soil moisture content, pH in CaCl_2_, total carbon (C_tot_), inorganic C, and total nitrogen (N_tot_) contents were determined according to standard procedures as outlined in Online Resource 1, Supplementary Materials and Methods. Organic carbon (C_org_) was calculated as the difference between C_tot_ and inorganic C. In early June 2015, detailed physio-chemical analyses were conducted on soil samples derived from additional soil pits (*n* = 4) to characterize the habitat. The following parameters were determined: fine and coarse root biomass, stone fraction, bulk density, pH, C_org_, N_tot_, soil nutrients, and exchangeable ions (Online Resource 1, Table S1).

For DNA isolation from soil samples, 800 μl of the soil suspension in LifeGuard Soil Preservation Solution (see above) was transferred to the wells of a Bead Plate from the PowerSoil-htp 96-Well Soil DNA Isolation Kit (MO BIO, Carlsbad, CA, USA). After centrifugation (4000*g*, 15 min) and removal of the supernatant, the protocol of the manufacturer was followed with modifications (see Online Resource 1, Supplementary Materials and Methods). The fungal ITS2-region, which has been suggested as preferred barcoding region for fungal community analyses [44], was amplified with primer pair ITS3Mix/ITS4Mix (adapted from [37], for details see Online Resource 1, Supplementary Materials and Methods). Library preparation and Illumina MiSeq sequencing of fungal amplicons were conducted as described [45]. Illumina MiSeq PE250 sequencing was performed at the NGS Unit of the Vienna Biocenter Core Facility GmbH (Vienna, Austria). For details, see Online Resource 1.

### Data Evaluation and Statistical Analysis

Initial quality filtering was done with Trimmomatic v. 0.36 [46]. USEARCH (v. 9.0.2132) program suite [47] was used for merging the forward and reverse reads with a minimal overlap of 30 bp with fastq_mergepairs. Sequences < 280 bp were all of non-fungal origin and thus filtered out. Further steps essentially followed [45]. In brief, FASTX toolkit script fastx_barcode_splitter.pl was used to sort out project-specific fungal sequences; USEARCH scripts were used for chimera detection and filtering underrepresented sequences (< 10). VSEARCH [48] was used for clustering and counting sequences per cluster, using a 97% sequence similarity, which is a widely used threshold for the ITS region [e.g., 18] and lies between generally accepted limits for discrimination of species and genera [49]. The results were a sequence file of Operational Taxonomic Units (OTUs) holding one representative sequence per cluster and a table with counts of each OTU per sample. The OTU sequences were aligned using Clustal Omega, and a PhyML tree [50] was calculated. Taxonomic affiliation of OTUs was done with the UTAX script against the UNITE database [51]; manual editing increased phylogenetic accuracy [52]. Given the set sequence similarity threshold, species names provided for OTUs must be considered *sensu lato*. Non-fungal sequences were removed from further analyses. Ecological guild mapping of OTUs of the total fungal community (TOT) into three functional groups, i.e., saprotrophic (SAP), symbiotic (SYM), and potentially plant pathogenic (PAT) fungi, was based on scientific literature as described previously [53] (Online Resource 2, Table S2). The mapping was primarily conducted at the genus level and refined manually were necessary. Additionally, ectomycorrhizal morphotyping (A. Bittner, unpublished results) allowed mapping selected OTUs affiliated to the Hyaloscyphaceae as SYM. OTUs which could not be categorized were thus labeled “not assigned” (NA). Sequencing and associated data have been deposited at NCBI BioProject PRJNA521677, BioSamples SAMN12582230-SAMN12582341, and GenBank accession numbers MK626959-MK627467 (see also Online Resource 2, Table S2).

On average 67,875 fungal reads were obtained per sample. For all further calculations, samples were rarefied to 10,022 reads by the function Rarefy from R package “GUniFrac” [54]. No statistically significant differences were found for raw read numbers between seasons (*p* = 0.71) or plots (*p* = 0.12); observed OTU richness was independent of raw read numbers before rarefaction (*p* = 0.32). The observed richness in the number of fungal OTUs, the Berger-Parker index for the most abundant OTU, Simpson’s inverse diversity index (1/D), and Shannon’s diversity index (only shown for comparison with other studies, where no other diversity index is shown) were calculated for each single sample using R package “vegan” [55] (Online Resource 1, Figure S4). Additionally, to mimic pooling of individual soil samples, as practiced frequently by other studies, rarefied reads from 3 to 4 samples per plot were pooled by season and 1/D of the composite dataset was calculated.

Generalized UniFrac Distances (UF), taking phylogenetic information into account [56], were calculated using R package “vegan” [55]. Two additional indices for β-diversity, Morisita-Horn (MH) and Bray-Curtis (BC) [57], were calculated with the software EstimateS v.9.1 [58] for total fungal communities and functional guilds. Classical multidimensional scaling of the resulting data matrix was performed as principal coordinate analysis (PCoA) to obtain a 2-dimensional representation of dissimilarities [59]. For PCoA, the distances between fungal communities from separate samples were calculated as generalized UniFrac distances using the R package “GUniFrac” [54] with log-scaled data and an alpha value of 0.5 to avoid domination by overabundant species [56]. The “adonis” function [60] from R package “vegan” [55] was used to describe the significances of seasonal differences among the fungal communities.

Environmental distance (ED) for correlation analyses was calculated as the Euclidean distance from differences in space (i.e., horizontal distance of coordinates), soil pH, and soil C_org_ similar to Goldmann et al. [61]. Details are provided in Online Resource 1, Supplementary Materials and Methods.

Random forest modeling was performed using the R package “randomForest” [62] as previously described [45]. Models were calculated with parameters season (i.e., sampling date), soil pH, and soil C_org_ as dependent and OTUs as independent variables. Models with the parameter “season” were calculated separately for TOT and SYM. A total of 2000 trees per model were calculated. During model building, the “out of the bag error” was recorded and the error rates (classification) or mean squared errors (regression) were used to estimate the importance of each OTU. Importance of single variables for correct classification is given as a mean decrease in accuracy (Online Resource 2, Table S2) (see package documentation for details [62]). The most important OTUs for inferring the sampling date from TOT and SYM are listed in Table S3 and S4, respectively (Online Resource 1). OTUs resulting in ≥ 7.5% mean decrease in accuracy for predicting soil pH or C_org_ are listed in Table S5.

Within the manuscript, means and standard deviation (mean ± SD) are given if not otherwise denoted.

## Results

### Fungal Diversity

In the studied mountain beech forest, 509 fungal OTUs (Online Resource 2, Table S2) were retrieved from 112 soil samples collected in May and August 2015; on average, 231±25 OTUs were found per sample (Online Resource 1, Figure S4a). The most abundant OTU (OTU_3) accounted for 5.8% of the total community. Maximum abundance was 19±12% per sample. Certain samples were, however, remarkably uneven with one highly dominant OTU, which could account for up to 75% of the total community (Figure S4b). The fungal community was highly diverse with an Inverse Simpson’s Diversity Index of 20±11 per sample (Figure S4c) and 32±12 for plots (i.e., pooled data of three to four single samples).

**Fig. 1:**
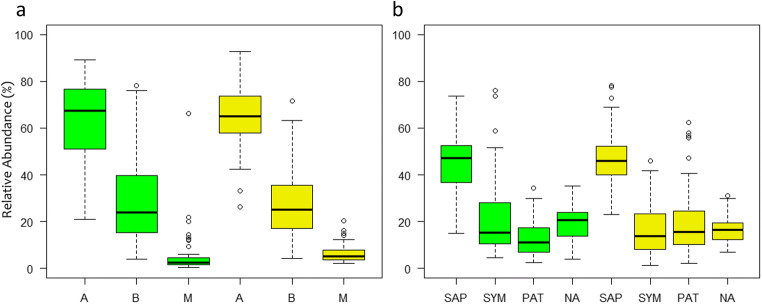
Relative abundance of fungal phyla (**a**) and ecological guilds (**b**) in May (green; spring) and August 2015 (yellow; summer) at the Molln site. A: Ascomycota; B: Basidiomycota; M: Mortierellomycota and Mucoromycota; phyla <1% are not displayed (see Online Resource 2, Table S2 for a list of OTUs). SAP, saprotrophic fungi; SYM, symbiotic fungi; PAT, potentially plant pathogenic fungi; NA, fungal guild not assigned

Both in May and August, the total fungal community (TOT) was dominated by Ascomycota followed by Basidiomycota, Mortierellomycota, and Mucoromycota (Fig. 1a). All other fungal phyla (e.g., Zoopagomycota and Chytridiomycota) possessed relative abundances <1% and/or were only occasionally detected. Interestingly, Glomeromycota, which can form arbuscular mycorrhizae with the roots of many plant species [28], were totally absent from the dataset, although some potential host plants were (sparsely) present at the study site as understorey vegetation (see Online Resource 1, Supplementary Materials and Methods). A list of detected fungal OTUs is provided in Online Resource 2, Table S2.

**Fig. 2:**
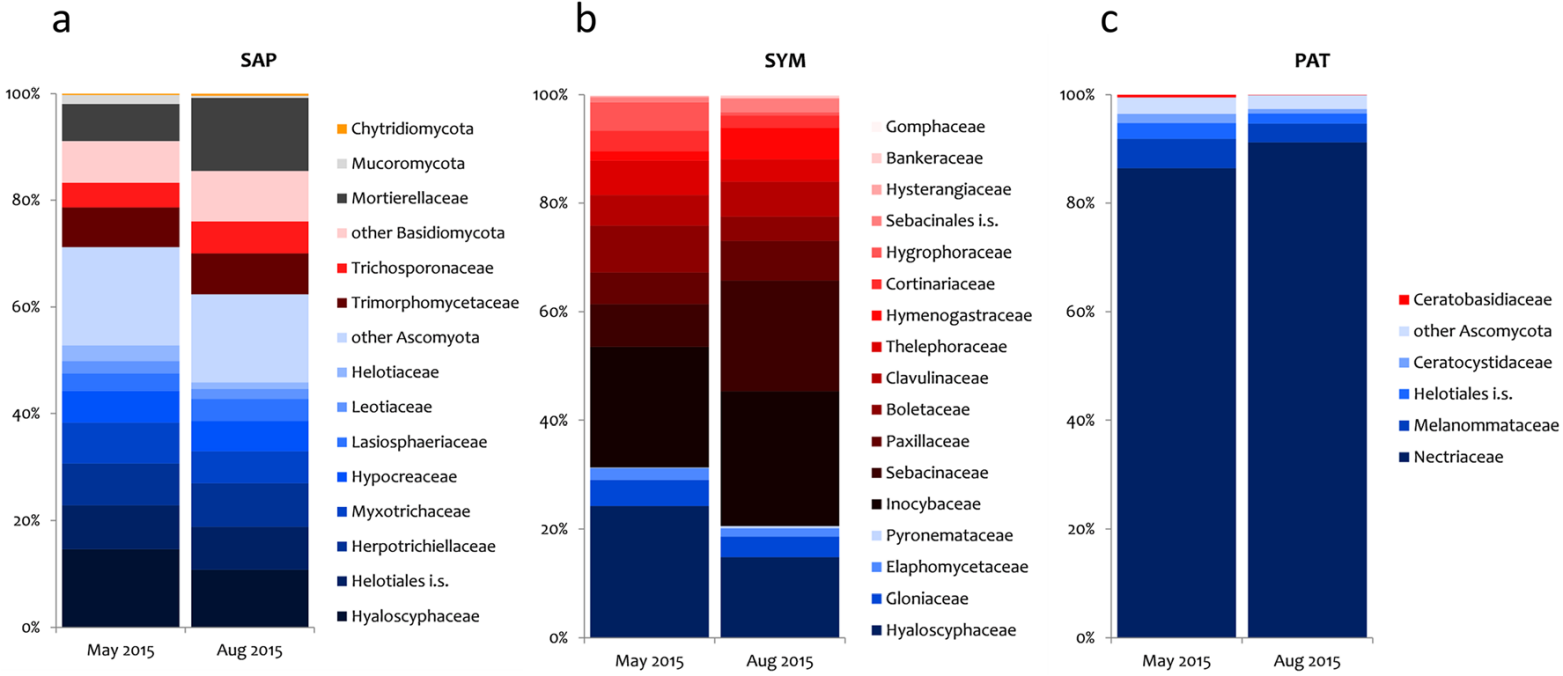
Relative abundance of fungal families in the three different ecological groups in May (spring) and August (summer) 2015 at the Molln site. **a** SAP, saprotrophs; **b** SYM, symbionts (mainly ECM and sebacinalean root interactions [63]); **c** PAT, potentially plant pathogenic fungi. Ascomycota are colored in blue, Basidiomycota in red, Mucoromycota and Mortierellomycota in black/grey, and Chytridiomycota in yellow

Saprotrophic fungi (SAP) composed the dominant guild (215 OTUs; 46.6±11.7% of reads per sample; Fig. 1b)—constituted by Hyaloscyphaceae, basidiomycetous yeasts, and Mortierellaceae (Fig. 2). The symbiotic fungi (SYM; 100 OTUs; 18.8±13.4%) were not only dominated by families known to be ectomycorrhizal (ECM; in particular Inocybaceae, Hyaloscyphaceae) but also contained taxa from the Sebacinales, which can form ectomycorrhizas and other root interactions [63]; interestingly, no Russulaceae were found. Potentially, plant pathogenic fungi (PAT) occurred at relatively high abundances (34 OTUs; 16.4±12.0%), especially species from the nectriaceous genera *Dactylonectria*, *Ilyonectria*, and *Neonectria*. A high proportion of sequences could not be assigned to any ecological guild (“NA”; 160 OTUs; 18.2±6.3%). This includes the most abundant OTU_3, which has an unclear affiliation in the Leotiomycetes with a close relationship to the Pseudeurotiaceae.

### Seasonal Changes

**Fig. 3:**
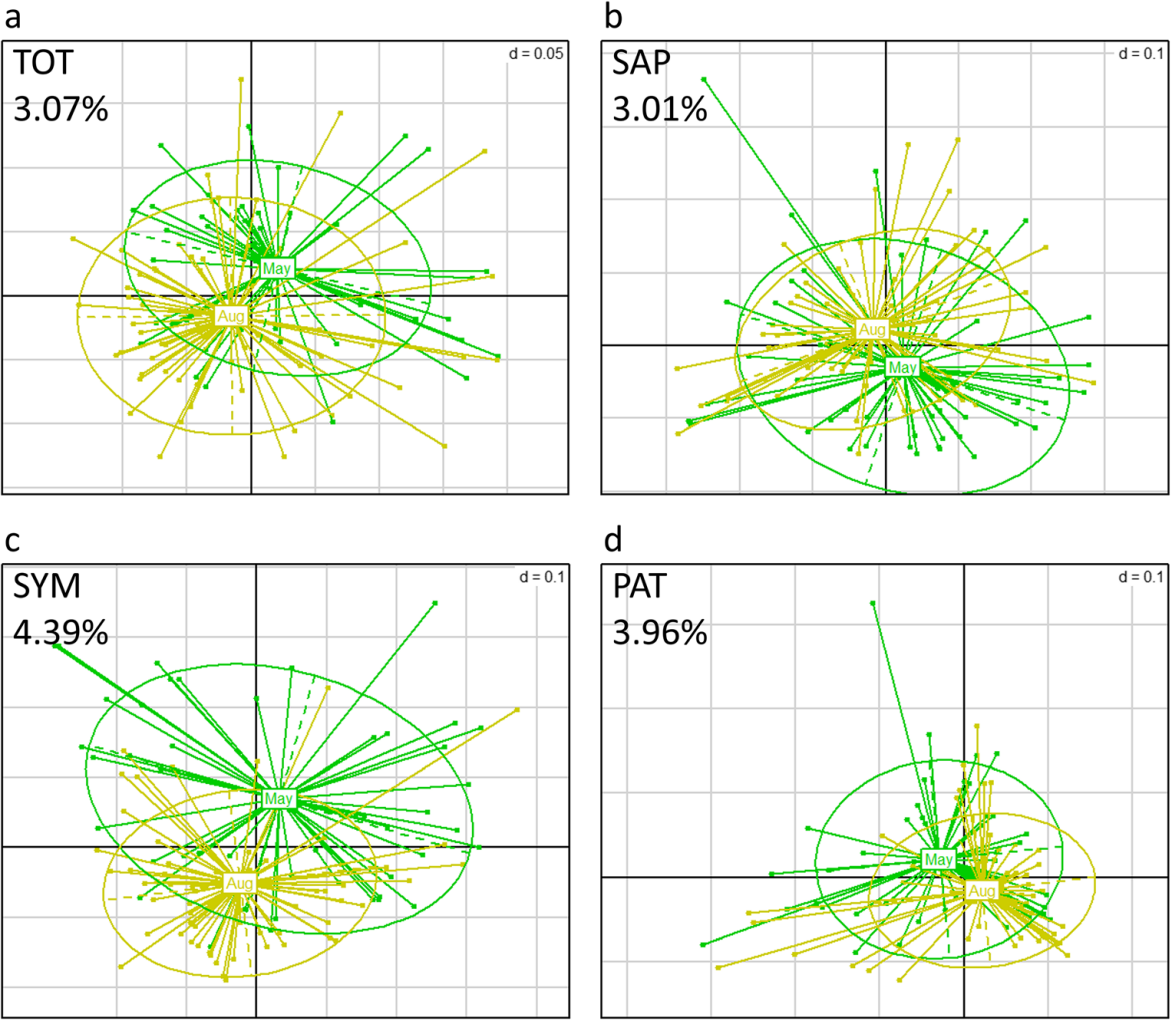
Seasonal changes in fungal community composition at the Molln site. PCoA of log-transformed UniFrac distances (green: May (spring) 2015; yellow: August (summer) 2015) of the total fungal community, TOT (**a**); the saprotrophic fungi, SAP (**b**); the symbiotic fungi, SYM (**c**); and the potentially plant pathogenic fungi, PAT (**d**). For scaling of grids, see ***d*** value in the upper right corner of each subpanel

Comparing May (spring) and August (late summer) 2015, no major differences were observed in relative abundances of the phyla Ascomycota, Basidiomycota, Mortierellomycota, or Mucoromycota (Fig. 1a) and of the ecological guilds (Fig. 1b). Only a minor shift in the total fungal composition (TOT) in response to season was observed (3.07% of variance explained by season in Adonis; Fig. 3a); the most important OTUs from TOT for inferring the season are listed in Online Resource 1, Table S3. The majority of OTUs with a high importance for explanation of seasonal variations belongs to the so-called basal fungal lineages, more specifically to the Mortierellomycota and the Chytridiomycota. Relative abundances of single OTUs with significant seasonal changes did, however, not exceed 1%, whereas dominant groups in the fungal community showed less pronounced seasonal shifts.

**Fig. 4:**
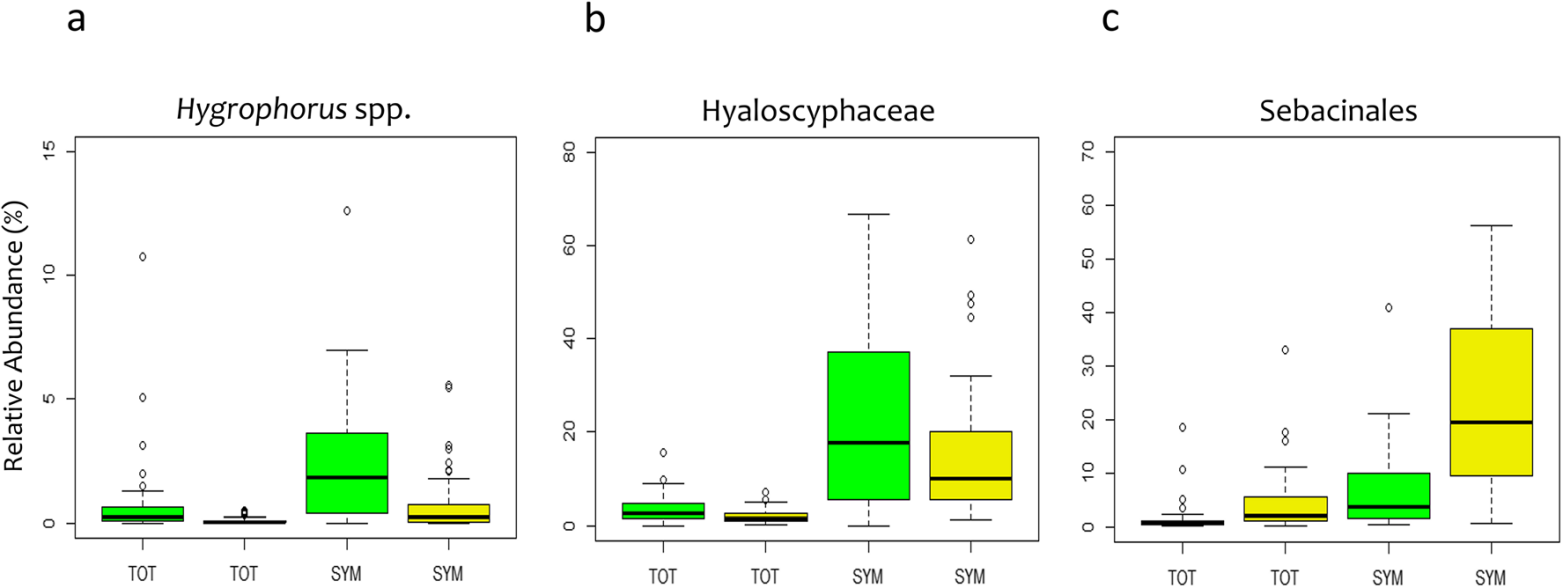
Seasonal responses of selected taxa of mycorrhizal fungi at the Molln site. *Hygrophorus* spp. (i.e., OTU_96 and 125) (**a**), Hyaloscyphaceae (i.e., OTU_11 and 27) (**b**), and Sebacinales (26 different OTUs) (**c**). Data are shown for the relative abundance in the total fungal community (TOT) and in the subset of symbiotic (SYM) fungi in May (green; spring) and August 2015 (yellow; summer). Please note the different scales of ***y***-axes

No major effect of season on the relative abundances of ecological guilds was observed (Fig. 1b). Separate analyses of the ecological guilds indicated that the community compositions of SYM and PAT showed slightly more pronounced seasonal shifts (4.39% and 3.96%, respectively) than those of SAP (3.01%) (Fig. 3b-d); underlying taxa could be identified (Online Resource 1, Table S4). *Hygrophorus* spp. (i.e., OTU_96 and 125) and ectomycorrhizal Hyaloscyphaceae (i.e., OTU_11 and 27) were among key taxa explaining the seasonal shift in SYM composition—both taxa being less abundant in August compared to May 2015 (Fig. 4a, b). Most OTUs affiliated to the Sebacinales had a greater abundance in August, rendering the seasonal difference between combined sebacinalean OTUs significant (Fig. 4c).

### Spatial Heterogeneity and Environmental Influences

Soils at the Molln experimental site showed differences in C_org_, N_tot_, soil moisture, and pH (Online Resource 1, Figure S5). Contents of C_org_ and N_tot_ were highly correlated, with a relatively constant C:N ratio of 14.8±1.3. Therefore, the effects of N_tot_ could not be separated from the effects of C_org_. Gravimetric soil moisture was generally greater in May compared to August and was strongly correlated to C_org_ within each season (Online Resource 1, Figure S5b).

**Fig. 5:**
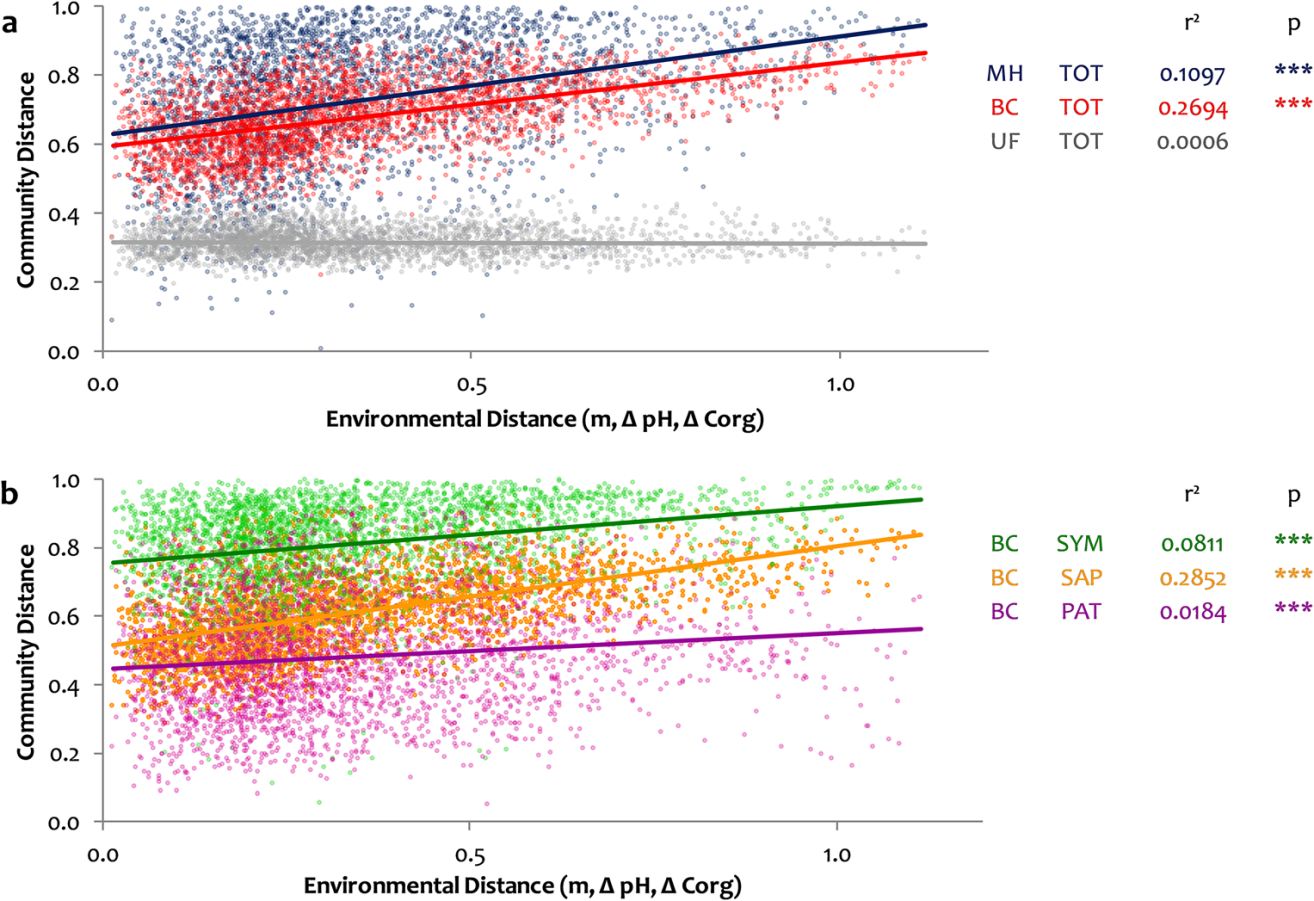
Environmental influences on fungal β-diversity at the Molln site. **a** Indices of β-diversity, Morisita-Horn (MH, blue), Bray-Curtis (BC, red), and UniFrac (UF, grey), were calculated from the fungal community data and plotted against the environmental distance. **b** BC β-diversity of symbiotic (SYM, green), saprotrophic (SAP, orange), and potentially plant pathogenic (PAT, purple) fungal guilds. Regression coefficients (***r***^2^) and significance levels (***p*** < 0.001) are shown in corresponding colors

Organic carbon had a minor effect on fungal β-diversity (6.5% of the variation of the Bray-Curtis distance – BC), a stronger effect was found for soil pH (17.3% of the variation of BC, Online Resource 1, Figure S6). Geographic distance alone could explain 16.4% of the variation of BC. Combining geographic distance and differences in pH and C_org_ into an environmental distance (ED) index explained 26.9% of total soil fungal BC β-diversity (Fig. 5a). ED was significantly related to BC β-diversity per guild (SAP, SYM, and PAT; Fig. 5b). SYM had the greatest β-diversity, PAT the least; in both guilds, BC slightly increased with ED; β-diversity of SAP, on the other hand, increased more strongly with ED. A substantially higher fraction of the variance was explained by ED for the SAP guild (28.5%) than for SYM (8.1%) or PAT (1.8%).

**Fig. 6:**
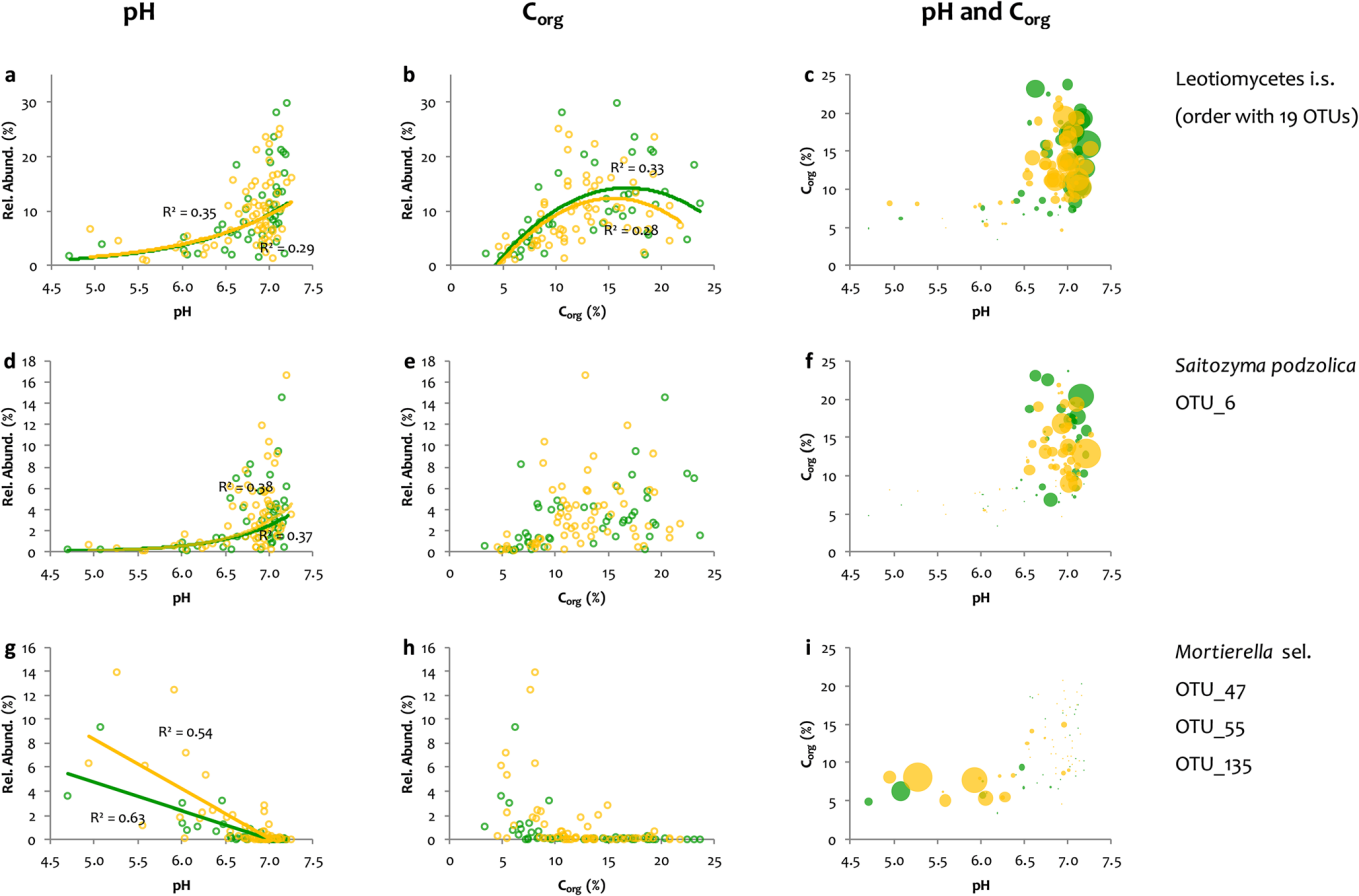
Relative abundances (%) of selected fungal taxa in response to soil pH (**a**, **d**, **g**), soil organic C content (C_org_) (**b**, **e**, **h**), and both factors (**c**, **f**, **i**) at the Molln site. Samples from May (green; spring) and August 2015 (yellow; summer) are shown separately. Where meaningful, regression lines were drawn (**a** Leotiomycetes *i.s.* pH, exponential; **b** Leotiomycetes *i.s.* C_org_, polynomial; **d** *Saitozyma podzolica* OTU 6, exponential; **g** *Mortierella* sel. pH, linear; all shown correlations are highly significant at ***p*** < 0.001). In the response diagrams of fungal taxa to both pH and C_org_ (**c**, **f**, **i**), the relative abundance is pictured by the diameter of the circles. The largest circles indicate 29.7% relative abundance for Leotiomycetes *i.s.* (**c**), 16.6% for *Saitozyma podzolica* OTU 6 (**f**), and 13.9% for *Mortierella* sel. (**i**)

Although no significant influence of soil pH or C_org_ on the relative abundances of the phyla (Ascomycota or Basidiomycota) or the fungal guilds occurred, both parameters had significant effects on specific, abundant OTUs—dominantly related to SAP or NA guilds (Online Resources 1, Table S5). A group of fungi in the Leotiomycetes (affiliated to the families Pseudeurotiaceae and Myxotrichaceae) increased in relative abundance from 1.5% at pH 4.7 to 29.7% at pH 7.2 (Fig. 6a); rel. abundances >20% were found at C_org_ contents of 10-20% (Fig. 6b), which occurred at pH >6.5 (Fig. 6c). Similar, the basidiomycetous yeast *Saitozyma podzolica* (OTU_6) varied in relative abundances between <1.5 at pH <6.5 and C_org_ <6.5% (Fig. 6d, e) to a relative abundance of 16.6% at pH = 7.2 and C_org_ = 12.9% (Fig. 6f). In contrast, greater abundances in soils with a pH of roughly <6.5 and C_org_ <10% were observed for three OTUs from the genus *Mortierella*, i.e., *M.* aff. *elongata* (OTU_47), *M. pseudozygospora* (OTU_55), and *Mortierella* sp. (OTU_135; Fig. 6g h, i). The ECM fungus *Suillellus luridus* tended to occur in samples with a pH >6.8 (data not shown); while holding a maximum abundance of 74% in a single sample, *S. luridus* was generally a rare species (Online Resources 2, Table S2).

## Discussion

### Fungal Community and Diversity

Several studies focused on soil fungal diversity in beech forest stands, often with a specific emphasis on mycorrhizal fungi [e.g 18, 64, 65]. The sites used in these studies were mainly characterized by acidic soils and situated in the lowlands. In contrast, the Molln experimental site is situated on a steep south-facing slope in the Austrian Northern Calcareous Alps, featuring shallow soils with pH values ranging predominantly from 6.5 to 7.3 (total range 4.7-7.3), and high Ca contents (~13 mg g^-1^; Table S1) in the top mineral soil [8].

In agreement with our first hypothesis, we found a highly diverse fungal community in the studied mountain forest. At the experimental site, the median of observed richness was 231 OTUs per sample (Figure S4a). In contrast, beech forest stands in Northern, Central, and Southern Germany were reported to hold an observed soil fungal richness of <180 OTUs per sample and featured inversed Simpson’s indices <20 (for pooled samples) [18]. Pooling of sequence data from three (May 2015) or four (August 2015) soil samples at our mountainous beech forest stand resulted in inverse Simpson’s indices of 26 and 34 in May and August, respectively. This difference might be partially attributed to the use of different barcoding regions used for fungal community profiling, i.e., the whole ITS region by Wubet et al. [18] vs. the ITS2 region in our study. However, a greater fungal diversity at near neutral soil pH is in agreement with recent findings, where fungal diversity responded in a unimodal relationship to soil pH with a peak around pH 6 [66]. Similarly, a strong positive influence of soil Ca content on the richness of fungi was reported earlier [37]. Both point toward a more diverse soil fungal community in beech stands on calcareous bedrock compared to beech forests with more acidic soil conditions. Other potential effects of soil parameters are discussed below.

The horizontal spatial heterogeneity of the fungal community within a maximum geographic distance of ~700 m was very high; three different indices of β-diversity were applied to examine the dissimilarities (Online Resource 1, Figure S6a). The Bray-Curtis index (BC) possessed intermediate, and the UniFrac distance (UF) low dissimilarities, pointing towards a highly conserved phylogenetic composition of the fungal community. In contrast, the high Morisita-Horn index (MH) values indicate pronounced changes in abundance of dominant OTUs at short distances (<10 m) potentially related to the stands’ heterogeneous soil topography.

The fungal community at the Molln experimental site was remarkably distinct compared to those reported earlier. Specifically, Ascomycota was more frequent than Basidiomycota at the studied site (Fig. 1a). Most previous studies, including those conducted in lowland beech forests, reported higher frequencies of Basidiomycota or at least a balanced ratio of both phyla [18, 37, 61, 64, 67–69]. While the prevalence of Ascomycota was thus considered untypical for soil of temperate forest in general and beech forest in specific, our study indicates that this assumption might not hold true for mountainous beech stands on calcareous bedrock.

As many basidiomycetes are ECM fungi, the relative abundance of ECM fungi was also low, accounting for <25% of the total soil fungal community in the vast majority of samples (Fig. 1b)—lower than reported for other European beech forests. Temperate forest soils are generally dominated by ECM fungi [e.g., 70] and published data on relative ECM abundances in (lowland) beech forest soils range from 32 [67] to 90% [68]. The observed differences might be related to distinct environmental factors of the studied beech stand (as discussed below). A combination of soil factors, which favor saprotrophic fungi over symbiotic fungi, has probably contributed to low ECM abundance in soil. Beech root colonization by ECM fungi has previously been described to be higher [71] or lower [72] on calcareous soils with near-neutral pH compared to siliceous soils with acidic pH. The use of different primer target regions for fungal community profiling in different studies could have partially contributed to the observed differences. Op De Beeck et al., however, reported that using e.g. either ITS1 vs. ITS2 regions to determine the relative abundances of Ascomycota and Basidiomycota had a relatively minor effect [73].

Among ECM fungi, the genus *Russula* is one of the most broadly distributed worldwide [74]. As Russulaceae were common among the dominant members of the ECM community of beech stands [18, 61, 64, 67–69], the complete absence of Russulaceae from the ECM community of the studied mature mountainous beech stand was highly surprising. A strong bias by the amplification protocol cannot be expected, as no mismatches of the primers were found for representative sequences of Russulaceae in the NCBI GenBank. Furthermore, Russulaceae were regularly found in soil samples from other temperate deciduous forests with the original primers [37] (for visualization see Fig. 77 in [24]) and in a parallel study with the same amplification and sequencing approach as applied herein (M. Gorfer, unpublished results). As a decline in *Russula* sp. abundance with increasing pH and decreasing C:N ratio was previously reported by others [18, 66, 75, 76], we speculated that the absence of *Russula* spp. at the site may be (partially) related to the near-neutral pH and a low C:N ratio [77], as discussed below in detail. Negative priority effects, i.e., pioneer fungal species reducing the subsequent colonization of a host by additional species, could have also contributed to the observed patterns—as previously shown for both ECM and AM colonization [78] and references within]. In addition, it has been suggested that long-distance spore dispersal is generally limited for *Russula* spp. [74]—potentially hampering spore dispersal particularly in a landscape dominated by mountain-valley systems and complex wind systems. Apart from the absence of Russulaceae, the ECM fungal community was similar to other beech-dominated forest stands, i.e., exhibiting relatively high abundances of *Inocybe* spp. and Sebacinales (Figure 2b, see [68] for comparison). A preference for high pH and low C:N ratios was previously described for *Inocybe* spp. and *Sebacina* spp. [18, 79] but also for *Suillellus luridus* [80] which however, was a rare species at the studied site.

In contrast to symbiotic fungi, high abundances of potentially plant pathogenic fungi were observed at the study site (Fig. 1b). On a global scale, soil fungal communities in temperate deciduous forest harbor <5% phytopathogenic fungi [37], while relative abundances of >15% were found at our study site. The potentially plant pathogenic community consisted mostly of taxa from the nectriaceous genera *Dactylonectria*, *Ilyonectria*, and *Neonectria—*causing root rot and other diseases in a wide variety of plants [81]. Protection of roots from pathogens by ECM fungi is well described; thus, an unfavorable ratio of symbiotic to plant pathogenic soil fungi could make mountain beech forests especially vulnerable to biotic stress. We can only speculate if the environmental conditions in harsher mountain forest habitats related to temperature extremes and restricted resource availability (i.e., shorter growing season and poor soil conditions) deem roots more susceptible to pathogenic fungi [82]. The topic of linkages between tolerance to both abiotic stress and potentially plant pathogenic fungi warrants further investigation, particularly addressing the rhizosphere of forest ecosystems on marginal sites.

### Seasonal Variation of Fungal Community Composition

A pronounced seasonality was reported recently for the fungal soil communities of temperate deciduous forests in China, with the diversity of the total fungal community being highest in July when trees possessed the most vigorous growth—potentially providing more C assimilate for fungal growth [32]. In our study, however, only subtle seasonal changes in the soil fungal community composition of the mountainous beech forest were found between spring (May) and late summer (August) 2015 (Figs. 1, 2, and 3). Species counts and β-diversity indices did not indicate major shifts in overall fungal community diversity (Online Resource 1, Figure S4). The abundance of fungal phyla (Fig. 1a) remained largely similar although some changes in specific taxa occurred. Those changes were generally more distinct not only in rare taxa, especially from the Mortierellomycota and the Chytridiomycota (both SAP; Table S3), but also in SYM-classified members of Hyaloscyphaceae and Sebacinales (Fig. 4c).

Based on previous findings, we hypothesized that taxa of the SYM guild, including Sebacinales with ectomycorrhiza-like root interactions, would be strongly affected by seasonal changes between spring and late summer [10, 32]. Recently fixed carbon is an important driver of soil biological processes [83], and C allocation belowground differs strongly with plant phenological and physiological states; thus, seasonal changes in the ECM community were previously linked to seasonal patterns in photosynthetic products and nutrient availability [84, 85]. In contrast, the richness of SAP fungi was earlier reported to be directly sensitive to soil moisture availability [37]. In partial agreement with our hypotheses, the SYM guild showed indeed a slightly more pronounced community response to season compared to the SAP guild, although overall seasonal differences in both guilds remained small (Fig. 3b-d). However, SYM-associated taxa with significantly greater abundances either in spring (*Hygrophorus* spp., Hyaloscyphaceae) or summer (Sebacinales) were identified (Fig. 4), indicating specific effects of different symbiotic taxa to seasonal patterns [84] but not generally greater abundances of SYM-associated taxa in summer (Fig. 1b). As storage products can play an important role in seasonal carbon allocation (e.g., spring vs. autumn) [86] and up to 12% of C in ECM fungi may originate from soil [87], we speculate that ECM taxa with a greater abundance in spring may rely less on recently assimilated C and are replaced by other symbiotic taxa such as Sebacinales later in the growing season. In addition, a different availability of soil nutrients in spring vs. late summer, driven by an increased uptake by roots and decreasing soil moisture availabilities until August, may underlie the pattern. In contrast to our findings, Voříšková et al. [31] and Štursová et al. [10] reported significantly greater relative abundances of several ECM fungal species in summer than in spring. We can only speculate that the less productive, deciduous mountainous stand at the Molln site provides less pronounced differences in seasonal C and nutrient availability belowground compared to previously studied (coniferous) forests with longer growth periods and greater resource availabilities.

Here, seasonal changes in relative abundance were generally more distinct in rare taxa, especially in taxa from the SAP-associated group Mortierellomycota and Chytridiomycota (Fig. 2a; Online Resource 1, Table S3). Three rare SAP-classified OTUs (relative abundance <1%) affiliated to the genus *Mortierella* showed greater relative abundances in (late) summer than in spring (Fig. 4b). *Mortierella* spp. are often classified as sugar fungi that as r-selected species can rapidly respond to transient nutrient peaks [88]. It is thus speculated that the observed higher relative abundance of selected *Mortierella* spp. in August might be caused by an increase in available nutrients by ongoing decomposition at sufficiently high soil moisture levels (Figure S5b; see [53, 88] for *Mortierella* spp., and [89] for Chytridiomycota’s soil moisture dependencies). Our results are in accordance with recent findings in a mountainous *Picea abies* forest, where the abundance of SAP-associated fungi in soil generally varied little between summer and spring, but *Mortierella*’s abundance increased in summer [10]. However, extreme weather events like extended drought spells, which were absent in the year of our study (Figure S1), are expected to induce more pronounced seasonal effects on soil fungal communities in general [90] and saprotrophs in particular [37]. Soil moisture at the Molln experimental site was lower in August than in May 2015, but was well above levels where drought stress would occur (Figure S5b). Seasonal differences in C availability to the soil fungal community thus warrant further investigation, particularly considering more parallel measurements of parameters underlying strong seasonal variation.

### Soil pH and Organic C Effects on Fungal Community

Our third hypothesis stated that the saprotrophic fungal community composition is primarily determined by soil properties. Soil chemistry in general, and soil pH in particular, has often been identified as a major parameter shaping soil fungal communities [37, 40, 66, 91], but see [92]. In accordance, the fungal β-diversity increased with environmental distance (ED) between sampling points, i.e., with combined changes in geographic distance, pH, and C_org_ contents (Fig. 5a). A substantial fraction of the variation remained, however, unexplained by the variables pH, C_org_, and geographic distance. Factors that were highly correlated to C_org_, i.e., N_tot_ and soil water content (Online Resource 1, Fig. S5a,b), could not be analyzed separately; soil Ca and P concentrations—of potentially predictive value [37]—were unfortunately not measured at the sample level. As hypothesized, ED explained a substantially higher fraction of the variability in BC diversity for SAP than for PAT or SAM guilds (Fig. 5b). Differences in soil pH and geographical distance alone could partially explain the increase in fungal β-diversity across the site, while C_org_ was a weaker predictor of β-diversity (Online Resource 1, Figure S6).

The gradients in pH and C_org_ allowed an identification of taxa, predominantly of the SAP guild and NA taxa, that showed strong responses to soil parameters (Table S5, Fig. 6). An abundant group of OTUs classified as Leotiomycetes i.s were preferentially found at mineral soil locations with a pH >6.5 and C_org_=10-20% (Fig. 6a-c). Interestingly, the genus *Oidiodendron* (Myxotrichaceae, Leotiomycetes i.s.) was shown by others to have a preference for acidic soils [93] but occurred at low abundance in more acidic parts of the studied site. We can only speculate that the abundance of *Oidiodendron* species is co-determined by the presence of intermediate to high C_org_ contents as lower pH values were correlated with lower C_org_ values at our study site (Fig. 6c; Online Resource 1, Figure S5c). Selected OTUs from *Mortierella* showed a preference for soil pH <6.5 and an associated C_org_ <10% (Fig. 6 g, h). Mortierellaceae were previously shown to rapidly colonize organic materials with preferred utilization of non-cellulosic C sources [88]. The preference for lower C_org_ contents was thus surprising, but might be driven by pH preferences. Soil pH and C_org_ had pronounced effects on rather abundant taxa, which were mostly classified as saprotrophic. Response curves to changes in pH and C_org_ were linear, exponential or polynomic (Fig. 6), which reflects different subsections of unimodal response curves often described for environmental factors [e.g., 94]. A strong influence of soil pH, which directly affects availability of major nutrients and toxic elements, on soil microbial community composition has been repeatedly reported [e.g., 37, 40]. Saprotrophic fungi, which largely depend on breakdown of complex organic matter through the activity of extracellular enzymes, are additionally affected by the pH optima of these enzymes.

The initially stated hypothesis that soil parameters primarily shape the SAP fungal community was thus supported by our results. A substantial fraction of the observed β-diversity remains, however, unexplained by the factors soil pH, C_org_, and geographic distance. It is thus assumed that additional factors including understorey vegetation, topographic heterogeneity, and further soil parameters such as Ca contents contribute to the observed high diversity.

### Conclusion

The studied mountain beech forest stand in the Northern Calcareous Alps of Austria showed a distinct and highly diverse soil fungal community with comparatively low relative abundances of basidiomycetes and of ECM fungi, while potentially plant pathogenic fungi were more prevalent than in previous studies (in lowland beech forests). Seasonal differences between May and August were minor and influenced mainly rare taxa. Soil properties like pH and C_org_ affected fungal community composition, affecting particularly the distribution pattern of dominant taxa and shaping the saprotrophic community.

## Supplementary Information

ESM 1 (PDF 2026 kb)

ESM 2 (XLSX 112 kb)

## Data Availability

Sequencing and associated data have been deposited at NCBI BioProject PRJNA521677, BioSamples SAMN12582230-SAMN12582341, and GenBank accession numbers MK626959-MK627467. Environmental data will be made available upon request.

## References

[1] Price M, Gratzer G, Duguma LA, Kohler T, Maselli D (2011) Mountain forests in a changing world: realizing values, addressing challenges. Food and Agriculture Organization of the United Nations (FAO), Rome

[2] Kräuchi N, Brang P, Schönenberger W (2000). Forests of mountainous regions: gaps in knowledge and research needs. For Ecol Manag.

[3] Bebi P, Seidl R, Motta R, Fuhr M, Firm D, Krumm F, Conedera M, Ginzler C, Wohlgemuth T, Kulakowski D (2017). Changes of forest cover and disturbance regimes in the mountain forests of the Alps. For Ecol Manag.

[4] Ellenberg H, Leuschner C (2010). Vegetation Mitteleuropas mit den Alpen.

[5] Dannenmann M, Bimüller C, Gschwendtner S, Leberecht M, Tejedor J, Bilela S, Gasche R, Hanewinkel M, Baltensweiler A, Kögel-Knabner I, Polle A, Schloter M, Simon J, Rennenberg H (2016) Climate change impairs nitrogen cycling in European beech forests. PLoS One 11:e0158823. 10.1371/journal.pone.0158823 10.1371/journal.pone.0158823 PMC4943676 27410969

[6] Simon J, Dannenmann M, Pena R, Gessler A, Rennenberg H (2017). Nitrogen nutrition of beech forests in a changing climate: importance of plant-soil-microbe water, carbon, and nitrogen interactions. Plant Soil.

[7] Reger B, Göttlein A, Katzensteiner K, Ewald J (2015). Assessing the sensitivity of mountain forests to site degradation in the Northern Limestone Alps, Europe. Mt Res Dev.

[8] BFW (1992) Österreichische Waldboden-Zustandsinventur. Ergebnisse. Waldbodenbericht. Österreichischer Agrarverlag, Vienna

[9] Katzensteiner K (2003) Effects of harvesting on nutrient leaching in a Norway spruce (*Picea abies *Karst.) ecosystem on a Lithic Leptosol in the Northern Limestone Alps. Plant Soil 250:59–73. 10.1023/a:1022821913932

[10] Štursová M, Kohout P, Human ZR, Baldrian P (2020) Production of fungal mycelia in a temperate coniferous forest shows distinct seasonal patterns. J Fungi 6:190. 10.3390/jof6040190 10.3390/jof6040190 PMC7712845 32993121

[11] Baruck J, Nestroy O, Sartori G, Baize D, Traidl R, Vrščaj B, Bräm E, Gruber FE, Heinrich K, Geitner C (2016). Soil classification and mapping in the Alps: the current state and future challenges. Geoderma.

[12] Tateno R, Takeda H (2003). Forest structure and tree species distribution in relation to topography-mediated heterogeneity of soil nitrogen and light at the forest floor. Ecol Res.

[13] Janssen P, Fuhr M, Bouget C (2018). Small variations in climate and soil conditions may have greater influence on multitaxon species occurrences than past and present human activities in temperate mountain forests. Divers Distrib.

[14] EEA (2010). Europe’s ecological backbone: recognising the true value of our mountains.

[15] Večeřa M, Divíšek J, Lenoir J, Jiménez-Alfaro B, Biurrun I, Knollová I, Agrillo E, Campos JA, Čarni A, Crespo Jiménez G, Ćuk M, Dimopoulos P, Ewald J, Fernández-González F, Gégout J-C, Indreica A, Jandt U, Jansen F, Kącki Z, Rašomavičius V, Řezníčková M, Rodwell JS, Schaminée JHJ, Šilc U, Svenning J-C, Swacha G, Vassilev K, Venanzoni R, Willner W, Wohlgemuth T, Chytrý M (2019). Alpha diversity of vascular plants in European forests. J Biogeogr.

[16] Kyaschenko J, Clemmensen KE, Karltun E, Lindahl BD (2017). Below-ground organic matter accumulation along a boreal forest fertility gradient relates to guild interaction within fungal communities. Ecol Lett.

[17] Sterkenburg E, Bahr A, Brandström Durling M, Clemmensen KE, Lindahl BD (2015). Changes in fungal communities along a boreal forest soil fertility gradient. New Phytol.

[18] Wubet T, Christ S, Schoning I, Boch S, Gawlich M, Schnabel B, Fischer M, Buscot F (2012). Differences in soil fungal communities between European beech (*Fagus sylvatica* L.) dominated forests are related to soil and understory vegetation. PLoS One.

[19] Baldrian P (2014). Distribution of extracellular enzymes in soils: spatial heterogeneity and determining factors at various scales. Soil Sci Soc Am J.

[20] Štursová M, Bárta J, Šantrůčková H, Baldrian P (2016) Small-scale spatial heterogeneity of ecosystem properties, microbial community composition and microbial activities in a temperate mountain forest soil. FEMS Microbiol Ecol 92:fiw185. https://doi.org/10.1093/femsec/fiw185 10.1093/femsec/fiw185 27604254

[21] Kobler J, Jandl R, Dirnböck T, Mirtl M, Schindlbacher A (2015). Effects of stand patchiness due to windthrow and bark beetle abatement measures on soil CO_2_ efflux and net ecosystem productivity of a managed temperate mountain forest. Eur J Forest Res.

[22] López-Mondéjar R, Brabcová V, Štursová M, Davidová A, Jansa J, Cajthaml T, Baldrian P (2018). Decomposer food web in a deciduous forest shows high share of generalist microorganisms and importance of microbial biomass recycling. ISME J.

[23] Gorfer M, Blumhoff M, Klaubauf S, Urban A, Inselsbacher E, Bandian D, Mitter B, Sessitsch A, Wanek W, Strauss J (2011). Community profiling and gene expression of fungal assimilatory nitrate reductases in agricultural soil. ISME J.

[24] Adamčík S, Looney B, Caboň M, Jančovičová S, Adamčíková K, Avis PG, Barajas M, Bhatt RP, Corrales A, Das K, Hampe F, Ghosh A, Gates G, Kälviäinen V, Khalid AN, Kiran M, De Lange R, Lee H, Lim YW, Kong A, Manz C, Ovrebo C, Saba M, Taipale T, Verbeken A, Wisitrassameewong K, Buyck B (2019) The quest for a globally comprehensible *Russula* language. Fungal Divers online first. 10.1007/s13225-019-00437-2

[25] Rosinger C, Sandén H, Matthews B, Mayer M, Godbold D (2018) Patterns in ectomycorrhizal diversity, community composition, and exploration types in European beech, pine, and spruce forests. Forests 9:445. 10.3390/f9080445

[26] van der Linde S, Suz LM, Orme CDL, Cox F, Andreae H, Asi E, Atkinson B, Benham S, Carroll C, Cools N, De Vos B, Dietrich H-P, Eichhorn J, Gehrmann J, Grebenc T, Gweon HS, Hansen K, Jacob F, Kristöfel F, Lech P, Manninger M, Martin J, Meesenburg H, Merilä P, Nicolas M, Pavlenda P, Rautio P, Schaub M, Schröck H-W, Seidling W, Šrámek V, Thimonier A, Thomsen IM, Titeux H, Vanguelova E, Verstraeten A, Vesterdal L, Waldner P, Wijk S, Zhang Y, Žlindra D, Bidartondo MI (2018) Environment and host as large-scale controls of ectomycorrhizal fungi. Nature 558:243–248. 10.1038/s41586-018-0189-9

[27] Nguyen NH, Song Z, Bates ST, Branco S, Tedersoo L, Menke J, Schilling JS, Kennedy PG (2016). FUNGuild: an open annotation tool for parsing fungal community datasets by ecological guild. Fungal Ecol.

[28] Smith S, Read D (2008) Mycorrhizal Symbiosis. 3rd ed. Academic Press, Cambridge

[29] Heinemeyer A, Hartley IP, Evans SP, Carreira De La Fuente JA, Ineson P (2007). Forest soil CO_2_ flux: uncovering the contribution and environmental responses of ectomycorrhizas. Glob Chang Biol.

[30] Pena R, Offermann C, Simon J, Naumann PS, Geßler A, Holst J, Dannenmann M, Mayer H, Kögel-Knabner I, Rennenberg H, Polle A (2010). Girdling affects ectomycorrhizal fungal (EMF) diversity and reveals functional differences in EMF community composition in a beech forest. Appl Environ Microbiol.

[31] Voříšková J, Brabcová V, Cajthaml T, Baldrian P (2014). Seasonal dynamics of fungal communities in a temperate oak forest soil. New Phytol.

[32] He J, Tedersoo L, Hu A, Han C, He D, Wei H, Jiao M, Anslan S, Nie Y, Jia Y, Zhang G, Yu G, Liu S, Shen W (2017) Greater diversity of soil fungal communities and distinguishable seasonal variation in temperate deciduous forests compared with subtropical evergreen forests of eastern China. FEMS Microbiol Ecol 93:fix069. 10.1093/femsec/fix069 10.1093/femsec/fix069 28854678

[33] Põlme S, Bahram M, Yamanaka T, Nara K, Dai YC, Grebenc T, Kraigher H, Toivonen M, Wang P-H, Matsuda Y, Naadel T, Kennedy PG, Kõljalg U, Tedersoo L (2013). Biogeography of ectomycorrhizal fungi associated with alders (*Alnus* spp.) in relation to biotic and abiotic variables at the global scale. New Phytol.

[34] Kohout P, Charvátová M, Štursová M, Mašínová T, Tomšovský M, Baldrian P (2018). Clearcutting alters decomposition processes and initiates complex restructuring of fungal communities in soil and tree roots. ISME J.

[35] Peršoh D, Borken W (2017). Impact of woody debris of different tree species on the microbial activity and community of an underlying organic horizon. Soil Biol Biochem.

[36] Guerreiro MA, Brachmann A, Begerow D, Peršoh D (2018). Transient leaf endophytes are the most active fungi in 1-year-old beech leaf litter. Fungal Divers.

[37] Tedersoo L, Bahram M, Põlme S, Kõljalg U, Yorou NS, Wijesundera R, Villarreal Ruiz L, Vasco-Palacios AM, Thu PQ, Suija A, Smith ME, Sharp C, Saluveer E, Saitta A, Rosas M, Riit T, Ratkowsky D, Pritsch K, Põldmaa K, Piepenbring M, Phosri C, Peterson M, Parts K, Pärtel K, Otsing E, Nouhra E, Njouonkou AL, Nilsson RH, Morgado LN, Mayor J, May TW, Majuakim L, Lodge DJ, Lee SS, Larsson KH, Kohout P, Hosaka K, Hiiesalu I, Henkel TW, Harend H, Guo LD, Greslebin A, Grelet G, Geml J, Gates G, Dunstan W, Dunk C, Drenkhan R, Dearnaley J, De Kesel A, Dang T, Chen X, Buegger F, Brearley FQ, Bonito G, Anslan S, Abell S, Abarenkov K (2014). Fungal biogeography. Global diversity and geography of soil fungi. Science.

[38] Okubara PA, Paulitz TC (2005) Root defense responses to fungal pathogens: a molecular perspective. Plant Soil 274:215–226.10.1007/s11104-004-7328-9

[39] Treseder KK, Marusenko Y, Romero-Olivares AL, Maltz MR (2016) Experimental warming alters potential function of the fungal community in boreal forest. Glob Chang Biol 22:3395–3404. 10.1111/gcb.13238 10.1111/gcb.13238 26836961

[40] Glassman SI, Wang IJ, Bruns TD (2017). Environmental filtering by pH and soil nutrients drives community assembly in fungi at fine spatial scales. Mol Ecol.

[41] ÖBf AG (2017) Bestandesbeschreibung FB 174 Forstbetrieb Steyrtal. Österreichische Bundesforste AG, Purkersdorf

[42] IUSS Working Group WRB (2006) World Reference Base for Soil Resources 2006: a Framework for International Classification, Correlation and Communication (2nd edition), Food and Agriculture Organization of the United Nations, Rome

[43] Finn GA, Straszewski AE, Peterson V (2007). A general growth stage key for describing trees and woody plants. Ann Appl Biol.

[44] Tedersoo L, Anslan S, Bahram M, Põlme S, Riit T, Liiv I, Kõljalg U, Kisand V, Nilsson H, Hildebrand F, Bork P, Abarenkov K (2015). Shotgun metagenomes and multiple primer pair-barcode combinations of amplicons reveal biases in metabarcoding analyses of fungi. MycoKeys.

[45] Keiblinger KM, Schneider M, Gorfer M, Paumann M, Deltedesco E, Berger H, Jochlinger L, Mentler A, Zechmeister-Boltenstern S, Soja G, Zehetner F (2018). Assessment of Cu applications in two contrasting soils-effects on soil microbial activity and the fungal community structure. Ecotoxicology.

[46] Bolger AM, Lohse M, Usadel B (2014). Trimmomatic: a flexible trimmer for Illumina sequence data. Bioinformatics.

[47] Edgar RC (2010). Search and clustering orders of magnitude faster than BLAST. Bioinformatics.

[48] Rognes T, Flouri T, Nichols B, Quince C, Mahé F (2016). VSEARCH: a versatile open source tool for metagenomics. PeerJ.

[49] Vu D, Groenewald M, de Vries M, Gehrmann T, Stielow B, Eberhardt U, Al-Hatmi A, Groenewald JZ, Cardinali G, Houbraken J, Boekhout T, Crous PW, Robert V, Verkley GJM (2019). Large-scale generation and analysis of filamentous fungal DNA barcodes boosts coverage for kingdom fungi and reveals thresholds for fungal species and higher taxon delimitation. Stud Mycol.

[50] Guindon S, Gascuel O (2003). A simple, fast, and accurate algorithm to estimate large phylogenies by maximum likelihood. Syst Biol.

[51] Kõljalg U, Nilsson RH, Abarenkov K, Tedersoo L, Taylor AF, Bahram M, Bates ST, Bruns TD, Bengtsson-Palme J, Callaghan TM, Douglas B, Drenkhan T, Eberhardt U, Dueñas M, Grebenc T, Griffith GW, Hartmann M, Kirk PM, Kohout P, Larsson E, Lindahl BD, Lücking R, Martín MP, Matheny PB, Nguyen NH, Niskanen T, Oja J, Peay KG, Peintner U, Peterson M, Põldmaa K, Saag L, Saar I, Schüssler A, Scott JA, Senés C, Smith ME, Suija A, Taylor DL, Telleria MT, Weiss M, Larsson KH (2013). Towards a unified paradigm for sequence-based identification of fungi. Mol Ecol.

[52] Hofstetter V, Buyck B, Eyssartier G, Schnee S, Gindro K (2019). The unbearable lightness of sequenced-based identification. Fungal Divers.

[53] Deltedesco E, Keiblinger KM, Piepho H-P, Antonielli L, Pötsch EM, Zechmeister-Boltenstern S, Gorfer M (2020). Soil microbial community structure and function mainly respond to indirect effects in a multifactorial climate manipulation experiment. Soil Biol Biochem.

[54] Chen J (2018) Package ‘GUniFrac’. R package ver 1.1. https://cran.r-project.org/web/packages/GUniFrac/GUniFrac.pdf

[55] Oksanen J, Blanchet FG, Friendly M, Kindt R, Legendre P, McGlinn D, Minchin PR, O'Hara RB, Simpson GL, Solymos P, Stevens MHH, Szoecs E, Wagner H (2018) Package “vegan”. R Package ver 20.8. https://cranr-project.org/web/packages/vegan/vegan.pdf

[56] Chen J, Bittinger K, Charlson ES, Hoffmann C, Lewis J, Wu GD, Collman RG, Bushman FD, Li H (2012). Associating microbiome composition with environmental covariates using generalized UniFrac distances. Bioinformatics.

[57] Jost L, Chao A, Chazdon RL, Magurran AE, McGill BJ (2010). Compositional similarity and beta diversity. Biological diversity: frontiers in measurement and assessment.

[58] Colwell RK (2013) EstimateS: Statistical estimation of species richness and shared species from samples. Version 9. User’s Guide and application. http://purloclc.org/estimates

[59] Gower JC (1966). Some distance properties of latent root and vector methods used in multivariate analysis. Biometrika.

[60] Anderson MJ (2001). A new method for non-parametric multivariate analysis of variance. Austral Ecol.

[61] Goldmann K, Schroter K, Pena R, Schoning I, Schrumpf M, Buscot F, Polle A, Wubet T (2016). Divergent habitat filtering of root and soil fungal communities in temperate beech forests. Sci Rep.

[62] Liaw A, Wiener M (2002) Classification and regression by randomForest. R News 2:18–22. https://cran.r-project.org/doc/Rnews/Rnews_2002-3.pdf

[63] Weiss M, Waller F, Zuccaro A, Selosse MA (2016). Sebacinales - one thousand and one interactions with land plants. New Phytol.

[64] Uroz S, Oger P, Tisserand E, Cébron A, Turpault MP, Buée M, De Boer W, Leveau JH, Frey-Klett P (2016). Specific impacts of beech and Norway spruce on the structure and diversity of the rhizosphere and soil microbial communities. Sci Rep.

[65] Carrino-Kyker SR, Kluber LA, Petersen SM, Coyle KP, Hewins CR, DeForest JL, Smemo KA, Burke DJ (2016) Mycorrhizal fungal communities respond to experimental elevation of soil pH and P availability in temperate hardwood forests. FEMS Microbiol Ecol 92. 10.1093/femsec/fiw024 10.1093/femsec/fiw024 26850158

[66] Tedersoo L, Anslan S, Bahram M, Drenkhan R, Pritsch K, Buegger F, Padari A, Hagh-Doust N, Mikryukov V, Gohar D, Amiri R, Hiiesalu I, Lutter R, Rosenvald R, Rähn E, Adamson K, Drenkhan T, Tullus H, Jürimaa K, Sibul I, Otsing E, Põlme S, Metslaid M, Loit K, Agan A, Puusepp R, Varik I, Kõljalg U, Abarenkov K (2020) Regional-scale in-depth analysis of soil fungal diversity reveals strong pH and plant species effects in Northern Europe. Front Microbiol 11. 10.3389/fmicb.2020.01953 10.3389/fmicb.2020.01953 PMC7510051 33013735

[67] Bueé M, Reich M, Murat C, Morin E, Nilsson RH, Uroz S, Martin F (2009). 454 Pyrosequencing analyses of forest soils reveal an unexpectedly high fungal diversity. New Phytol.

[68] Nacke H, Goldmann K, Schoning I, Pfeiffer B, Kaiser K, Castillo-Villamizar GA, Schrumpf M, Buscot F, Daniel R, Wubet T (2016). Fine spatial scale variation of soil microbial communities under European beech and Norway spruce. Front Microbiol.

[69] Hartmann M, Lee S, Hallam SJ, Mohn WW (2009). Bacterial, archaeal and eukaryal community structures throughout soil horizons of harvested and naturally disturbed forest stands. Environ Microbiol.

[70] Shi L-L, Mortimer PE, Ferry Slik JW, Zou X-M, Xu J, Feng W-T, Qiao L (2014). Variation in forest soil fungal diversity along a latitudinal gradient. Fungal Divers.

[71] Leberecht M, Tu J, Polle A (2016). Acid and calcareous soils affect nitrogen nutrition and organic nitrogen uptake by beech seedlings (*Fagus sylvatica* L.) under drought, and their ectomycorrhizal community structure. Plant Soil.

[72] Wiemken V, Laczko E, Ineichen K, Boller T (2001). Effects of elevated carbon dioxide and nitrogen fertilization on mycorrhizal fine roots and the soil microbial community in beech-spruce ecosystems on siliceous and calcareous soil. Microb Ecol.

[73] Op De Beeck M, Lievens B, Busschaert P, Declerck S, Vangronsveld J, Colpaert JV (2014). Comparison and validation of some ITS primer pairs useful for fungal metabarcoding studies. PLoS One.

[74] Wang P, Zhang Y, Mi F, Tang X, He X, Cao Y, Liu C, Yang D, Dong J, Zhang K, Xu J (2015). Recent advances in population genetics of ectomycorrhizal mushrooms *Russula* spp. Mycology.

[75] Coince A, Cordier T, Lengellé J, Defossez E, Vacher C, Robin C, Buée M, Marçais B (2014). Leaf and root-associated fungal assemblages do not follow similar elevational diversity patterns. PLoS One.

[76] Toljander JF, Eberhardt U, Toljander YK, Paul LR, Taylor AFS (2006). Species composition of an ectomycorrhizal fungal community along a local nutrient gradient in a boreal forest. New Phytol.

[77] Cools N, Vesterdal L, De Vos B, Vanguelova E, Hansen K (2014). Tree species is the major factor explaining C:N ratios in European forest soils. For Ecol Manag.

[78] Johnson D (2015). Priorities for research on priority effects. New Phytol.

[79] Goldmann K, Schoning I, Buscot F, Wubet T (2015). Forest management type influences diversity and community composition of soil fungi across temperate forest ecosystems. Front Microbiol.

[80] Phillips R (1994) Mushrooms and Other Fungi of Great Britain and Europe. Macmillan, Oxford

[81] Chaverri P, Salgado C, Hirooka Y, Rossman AY, Samuels GJ (2011). Delimitation of *Neonectria* and *Cylindrocarpon* (Nectriaceae, Hypocreales, Ascomycota) and related genera with *Cylindrocarpon*-like anamorphs. Stud Mycol.

[82] Garbelotto M, Gonthier P (2017) Variability and disturbances as key factors in forest pathology and plant health studies. Forests 8:441. 10.3390/f8110441

[83] Heinonsalo J, Pumpanen J, Rasilo T, Hurme K-R, Ilvesniemi H (2010). Carbon partitioning in ectomycorrhizal Scots pine seedlings. Soil Biol Biochem.

[84] Nakayama M, Tateno R (2018). Solar radiation strongly influences the quantity of forest tree root exudates. Trees.

[85] Yin H, Wheeler E, Phillips RP (2014). Root-induced changes in nutrient cycling in forests depend on exudation rates. Soil Biol Biochem.

[86] Horwath WR, Pregitzer KS, Paul EA (1994). ^14^C Allocation in tree–soil systems. Tree Physiol.

[87] Hobbie EA, van Diepen LTA, Lilleskov EA, Ouimette AP, Finzi AC, Hofmockel KS (2014). Fungal functioning in a pine forest: evidence from a ^15^N-labeled global change experiment. New Phytol.

[88] Schmidt SK, Wilson KL, Meyer AF, Gebauer MM, King AJ (2008). Phylogeny and ecophysiology of opportunistic “snow molds” from a subalpine forest ecosystem. Microb Ecol.

[89] Freeman KR, Martin AP, Karki D, Lynch RC, Mitter MS, Meyer AF, Longcore JE, Simmons DR, Schmidt SK (2009). Evidence that chytrids dominate fungal communities in high-elevation soils. PNAS.

[90] Shi L, Guttenberger M, Kottke I, Hampp R (2002). The effect of drought on mycorrhizas of beech (*Fagus sylvatica* L.): changes in community structure, and the content of carbohydrates and nitrogen storage bodies of the fungi. Mycorrhiza.

[91] Fierer N, Strickland MS, Liptzin D, Bradford MA, Cleveland CC (2009). Global patterns in belowground communities. Ecol Lett.

[92] Rousk J, Bååth E, Brookes PC, Lauber CL, Lozupone C, Caporaso JG, Knight R, Fierer N (2010). Soil bacterial and fungal communities across a pH gradient in an arable soil. ISME J.

[93] Rice AV, Currah RS (2005). *Oidiodendron*: A survey of the named species and related anamorphs of *Myxotrichum*. Stud Mycol.

[94] Gryndler M, Šmilauer P, Šťovíček V, Nováková K, Hršelová H, Jansa J (2017). Truffle biogeography—a case study revealing ecological niche separation of different *Tuber* species. Ecol Evol.

