# Supplementary material for: High Fungal Diversity but Low Seasonal Dynamics and Ectomycorrhizal Abundance in a Mountain Beech Forest
Source: Microb Ecol. 2021 Mar 23;82(1):243–56. doi: 10.1007/s00248-021-01736-5 (PMC8282586; doi:10.1007/s00248-021-01736-5)
Supplement: Supplementary file 1 — (PDF 2026 kb) [file 248_2021_1736_MOESM1_ESM.pdf]

Online Resource 1

High Fungal Diversity but Low Seasonal Dynamics and Ectomycorrhizal Abundance in a Mountain Beech Forest

Markus Gorfer, Mathias Mayer, Harald Berger, Boris Rewald*, Claudia Tallian, Bradley Matthews, Hans Sandén, Klaus Katzensteiner, Douglas L. Godbold

***Correspondence:** Boris Rewald, Forest Ecology, University of Natural Resources and Life Sciences (BOKU), Peter-Jordan-Strasse 82, 1190 Vienna, Austria. Office: +43 1 47654 -91219, Fax: -91209, Email: boris.rewald@boku.ac.at

1 Supplementary Material and Methods

Vegetation, climate and weather at experiment site

The Molln experimental site is dominated by European beech (*Fagus sylvatica* L.) and sparsely intermixed by Norway spruce (*Picea abies* (L.) H. Karst), sycamore (*Acer pseudoplatanus* L.), European ash (*Fraxinus excelsior* L.), and silver fir (*Abies alba* Mill.). Tree diameter at breast height, tree height, tree density, and aboveground tree biomass were on average 34.2 cm, 23.7 m, 499 trees ha^{-1}, and 559 m^3 ha^{-1}, respectively. A sparse layer of understory trees from regeneration (beech, spruce and sycamore) was present. The ground vegetation cover consisted of a sparse layer of grasses (*Carex alba* Scop., *Carex sylvatica* Huds., *Brachypodium sylvaticum* (Huds.) Beauv.) and herbs (*Helleborus niger* L., *Galium odoratum* (L.) Scop., *Senecio ovatus* (G. Gaertn. & Al.) Willd., *Allium ursinum* L., *Mercurialis perennis* L.).

Average (1996-2011) annual air temperature and precipitation were 7.8°C and 1645 mm (data from nearest climate station, approx. 4 km apart), respectively [1]. Temperature and precipitation from January to August 2015 are provided in Figure S1.

Determination of soil parameters

In the laboratory, soil pH in soil:$CaCl_2$ (0.01 M) extracts was determined on fresh samples with a pH meter (pH735; WTW Inolab, Germany). Total carbon (C) and nitrogen (N) concentrations of 300 mg subsamples were measured with a TruSpec CN analyzer (Leco, St Joseph, USA) according to Austrian standard protocol ÖNORM L 1080 [2]; subsamples were ground and homogenized prior to

measurements. Inorganic C content was determined by the Scheibler method [3]. Organic C (C_{org}) content was determined as the difference of total and inorganic C contents.

In June 2015, 4 soil pits were excavated across the research site. Soil pits were dug down to bedrock. Humus and soil types were visually assessed [4,5]. The percentage fraction of fine roots (< 2 mm diameter), coarse roots (2 – 10 cm and > 10 cm diameter), and stones in the mineral soil was visually estimated on the soil pit wall in 10 cm increments (Table S1) [6]. Soil samples between 0 and 30 cm were taken by means of a soil corer (diameter: 5.6 cm, height: 4.2 cm). Due to a very high stone content, soil samples had to be taken with a spade below 30 cm. Note, only two soil pits were deeper than 30 cm. Soil samples were immediately brought to the laboratory under cooled conditions. In the laboratory, soil samples were passed through a 2 mm sieve. Soil moisture content was determined gravimetrically from each soil sample by drying a 20 g subsample at 105°C for 48 hours. Soil pH in soil:H_2O extracts and soil:$CaCl_2$ (0,01M) extracts was determined for fresh soil samples with a pH meter. Total carbon (C) and nitrogen (N) concentration of 300 mg subsamples were measured with a TruSpec CN analyzer (Leco Corp., St Joseph, MI, USA) according to ÖNORM L 1080 [2]. Subsamples were ground (Pulverisette 5; Fritsch, Germany) and homogenized prior to measurements. Inorganic C content of samples was determined by the Scheibler method [3]. The concentration of fifteen elements (Al, Ca, Cd, Cr, Cu, Fe, K, Mg, Mn, Na, Ni, P, Pb, S, Zn) was determined for 700 mg subsamples by means of the aqua regia digestion method [7] and a ICP-OES analyzer (Optima 8300, Perkin Elmer, Inc., Waltham, MA, USA). A barium chloride method was used to determine the exchangeable cations for the soil samples.

DNA isolation and amplification

For DNA isolation from soil samples, half of the suspension (= 800 µl) in LifeGuard Soil Preservation Solution was transferred to the wells of a Bead Plate from the PowerSoil-htp 96 Well Soil DNA Isolation Kit (MO BIO, Carlsbad, CA, USA). After centrifugation at 4000 g for 15 min and removal of the supernatant, the combined vacuum and centrifugation protocol of the manufacturer was followed. Cell lysis was done in a FastPrep-96 bead beater (MP Biomedicals, Santa Ana, CA, USA) twice at 6 m s^{-1} for 45 s with a 1 min. break before the second lysis. To increase recovery of DNA from soil [8], new Bead Solution and Solution C1 were added to the soil pellet after the first extraction (Ext. I) and the full extraction was repeated (Ext. II). Fungal community analysis was done separately for repeated extractions I and II from sampling in May 2015. The impact of sequential extraction on the fungal community composition was investigated in comparison to differences between plots. Following exclusion of too low sequence counts (<4000 /sample) and unreliable OTUs (not annotated as fungi or apparent in less than 5 samples) we normalized via rarefraction and calculated the unifraq distances (with abundance weights of alpha = 0.5) with R package GUniFrac [9] (see Figure S3). Using Permanova [R package vegan; 10] for distance based multivariate analysis of variance we calculated 4 different models and estimated the Akaike information criterion (AIC, based on the sequential sum of squares of the residuals as likelihoods) to find the optimal model. The optimal model (lowest AIC) is

based on plot alone. The hypothesis that the extraction-factor has no influence could be confirmed (F-test, $p>0.77$ for the extraction-factor for models where it was included). The hypothesis that the plot-factor has no influence could be rejected ($p<0.005$).

As no differences were detected, repeated extracts of August samples were combined before PCR. The fungal ITS2-region was amplified with primer pair ITS3Mix/ITS4Mix (adapted from [11], see below), which contain the Illumina Nextera adapters at their 5'-ends. Amplifications were done in quadruplicate with Phusion High Fidelity Polymerase in HF buffer (Thermo Scientific). PCR products quadruplicates were pooled and purified with Agencourt AMPure XP Beads (Beckman Coulter). Indexing was done with Nextera Index XT primers. After purification of indexed ITS2-amplicons with Agencourt AMPure XP Beads, PCR products were quantified with the Quant-iT dsDNA HS Assay Kit (Invitrogen) and pooled in equimolar amounts. Illumina MiSeq PE250 sequencing was performed at the NGS Unit of the Vienna Biocenter Core Facility GmbH (Vienna, Austria).

Primer details

Primer mixes based on original primers ITS3 and ITS4 [12] were used for amplification of the fungal ITS2 region from soil DNA samples. Primers contain fungal specific sequences at the 3' end (highlighted in bold, see below) and a 5'-tail for sequencing at the Illumina MiSeq platform as previously described [13]. Primers are composed of five different forward primers – ITS31-ITS35 as specified by [11] – and two different reverse primers – ITS4 [12] and ITS43S, a degenerate version of ITS4. Modifications from the originally described primer pair ITS3/ITS4 were introduced for improved coverage of the fungal kingdom including the taxa Chytridiomycota, Sebacinales, Glomeromycota, Sordariales and Archaeorhizomycetes [14]. Forward and reverse primers were separately mixed in equimolar ratios to obtain ITS3-Mix and ITS4-Mix, respectively.

ITS3Mix

ITS31_NeXTf	TCGTCGGCAGCGTCAGATGTGTATAAGAGACAG**CATCGATGAAGAACGCAG**
ITS32_NeXTf	TCGTCGGCAGCGTCAGATGTGTATAAGAGACAG**CAACGATGAAGAACGCAG**
ITS33_NeXTf	TCGTCGGCAGCGTCAGATGTGTATAAGAGACAG**CACCGATGAAGAACGCAG**
ITS34_NeXTf	TCGTCGGCAGCGTCAGATGTGTATAAGAGACAG**CATCGATGAAGAACGTAG**
ITS35_NeXTf	TCGTCGGCAGCGTCAGATGTGTATAAGAGACAG**CATCGATGAAGAACGTGG**

ITS4-Mix

ITS4_NeXTr	GTCTCGTGGGCTCGGAGATGTGTATAAGAGACAG**TCCTCCGCTTATTGATATGC**
ITS43S_NeXTr	GTCTCGTGGGCTCGGAGATGTGTATAAGAGACAG**TCCTSSSCTTATTGATATGC**

Data evaluation and statistical analysis

Regression analyses for correlation of community distance as a measure for β-diversity to environmental parameters were conducted with Daniel's XL Toolbox add-in for Excel, v.7.3.4 [15]. Environmental distance (ED) was calculated as Euclidean Distance from differences in space (horizontal distance from coordinates), pH and C_{org} similar to [16]. All differences were normalized to a maximum of 1. Geographic distance and difference in C_{org} were additionally multiplied by 0.5 to reflect the higher importance of pH as revealed by single factor regression analyses (see Figure S6). ED was thus calculated as:

$$ED = \sqrt{\left(\frac{0.5 \times \Delta m}{\Delta m_{max}}\right)^2 + \left(\frac{\Delta pH}{\Delta pH_{max}}\right)^2 + \left(\frac{0.5 \times \Delta Corg}{\Delta Corg_{max}}\right)^2}$$

where Δm is the distance in m between sampling points, Δm_{max} is the maximum distance in m between samples at the site, ΔpH is the difference in pH($CaCl_2$) between two soil samples, ΔpH_{max} is the maximum difference in soil pH at the site, $\Delta C_{org.}$ is the difference in $C_{org.}$ between two soil samples and $\Delta Corg_{max}$ is the maximum difference in soil C_{org} at the site.

For graphical representation of strongly responding taxa, phylogenetically related OTUs with similar responses to changes in pH and $C_{org.}$ were grouped (Figure 6). The group Leotiomycetes *i.s.* includes 19 OTUs affiliated to the families Pseudeurotiaceae and Myxotrichaceae (OTU 3, OTU 8, OTU 20, OTU 35, OTU 70, OTU 94, OTU 141, OTU 142, OTU 144, OTU 201, OTU 219, OTU 257, OTU 303, OTU 340, OTU 384, OTU 447, OTU 514, OTU 721, OTU 1135). The group of selected *Mortierella* species contains *M.* aff. *elongata* OTU 47, *M. pseudozygospora* OTU 55, and *Mortierella* sp. OTU 135. Selected taxa responding significantly to pH or $C_{org.}$ and having maximal abundances at favorable conditions of at least 10% are shown.

2 Supplementary Tables

Table S1 Soil parameters from the Molln research site in the Reichraminger Hintergebirge, Upper Austria (47°49'08" N, 14°23'34" E) (mean±(SE), $n_{0\text{-}30cm}$ = 4; $n_{30\text{-}50cm}$ = 2)

Soil parameter	Soil horizon				
	**0 – 10 cm**	**10 – 20 cm**	**20 – 30 cm**	**30 – 40 cm[a]**	**40 – 50 cm[a]**
Fine root fraction d < 2 mm (cm^2 cm^{-2})	40 (1.4)	12.8 (1.3)	4.6 (1.4)	3.6	1.3
Coarse root fraction 2 - 10 mm (cm^2 cm^{-2})	8.8 (1.7)	4.3 (0.5)	2.5 (0.9)	1.5	1
Coarse root fraction > 10 mm (cm^2 cm^{-2})	1.5 (0.3)	1.8 (0.6)	0.8 (0.3)	0.5	0
Stone fraction (cm^2 cm^{-2})	15.3 (5.6)	44 (13.2)	77 (15.1)	82.5	97
Bulk Density (g cm^{-3})	0.5 (0.08)	0.55 (0.09)	0.72 (0.2)	0.79	-
pH (H_2O)	7.26 (0.3)	7.7 (0.08)	8.03 (0.05)	7.89	7.85
pH (0.01M $CaCl_2$)	6.7 (0.19)	6.98 (0.07)	7.12 (0.05)	7.13	7.15
Total C (g g^{-1})	12.57 (3.46)	8.98 (2.41)	6.94 (2.03)	5.44	6.08
Total N (g g^{-1})	0.96 (0.21)	0.74 (0.17)	0.6 (0.17)	0.43	0.42
Inorganic C (g g^{-1})	0.15 (0.03)	0.29 (0.11)	0.69 (0.22)	0.83	1.73
Al (mg g^{-1})	43.51 (4.34)	49.55 (3.23)	51.64 (2.48)	50.51	47.06
Ca (mg g^{-1})	13.42 (3.33)	13.86 (2.84)	20.39 (6.15)	24.95	33.44
Cd (µg g^{-1})	1.41 (0.21)	1.32 (0.22)	1.15 (0.21)	0.99	0.95
Cr (µg g^{-1})	43.36 (3.39)	47.18 (2.31)	48.14 (1.79)	48.37	44.90
Cu (µg g^{-1})	15.32 (2.62)	14.8 (2.61)	14.25 (2.64)	14.82	13.72
Fe (mg g^{-1})	31.78 (1.63)	34.84 (0.71)	35.18 (0.24)	34.01	32.43
K (mg g^{-1})	6.33 (0.84)	7.13 (0.58)	7.77 (0.64)	8.52	7.90
Mg (mg g^{-1}	6.73 (0.52)	7.26 (0.39)	8.08 (0.59)	8.62	9.08
Mn (µg g^{-1})	792.44 (43.42)	800.77 (64.75)	816.43 (48.38)	570.78	583.26
Na (µg g^{-1})	203.27 (18.09)	221.37 (13.45)	251.02 (28.04)	245.30	243.07
Ni (µg g^{-1})	23.14 (1.37)	25.16 (0.77)	25.87 (1.28)	26.75	24.77
P (µg g^{-1})	661.61 (116.72)	574.84 (115.76)	564.77 (139.05)	575.09	500.77
Pb (µg g^{-1})	63.39 (8.11)	52.65 (8.63)	42 (7.94)	26.66	22.88
S (µg g^{-1})	801.61 (181.98)	657.39 (144.1)	572.69 (148.78)	444.22	442.86
Zn (µg g^{-1})	102.19 (12.55)	89.18 (10)	78.22 (11.49)	59.42	61.41
Exchang. Al (µg g^{-1})	0.79 [b]	0.15 [b]	[b]	[b]	[b]
Exchang. Ca (mg g^{-1})	6.78 (1.45)	6.29 (1.21)	5.92 (1.13)	4.76	4.88
Exchang. Fe (µg g^{-1})	0.43 (0.27)	0.23 (0.06)	0.16 (0.08)	0.08	0.26
Exchang. K (µg g^{-1})	97.64 (7.38)	85.47 (6.93)	83.14 (2.74)	80.57	83.64
Exchang. Mg (mg g^{-1})	1.27 (0.27)	1.25 (0.23)	1.14 (0.19)	1.02	1.03
Exchang. Mn (µg g^{-1})	21.62 (13.64)	4.5 [b]	0.13 [b]	[b]	0.09 [b]
Exchang. Na (µg g^{-1})	20.72 (1.96)	20.55 (2.02)	18.35 (0.97)	19.67	17.30

[a] Values derived from two soil pits only, [b] Measurements either below the detection limit of the analyzer or only one value available; no standard error of the mean was calculated for particular cases

Online Resource 2, Table S2 (see MS Excel file) List of retrieved fungal OTUs from the Molln site with information on the Genebank accession number; taxonomy according to Index Fungorum (www.indexfungorum.org), where classification at genus level was not possible higher level taxonomy was used instead; associated guilds (SAP: saprotrophic, SYM: symbiotic, PAT: potentially plant pathogenic, or NA: not assigned); partially ectomycorrhizal exploration type of SYM according to [17]; average abundances in percent for May (spring) and for August (summer) 2015; and relative importance (Mean Decrease in Accuracy) for random forest prediction of season, soil pH and soil C_{org}

Table S3 Most important OTUs for the prediction of sampling date, i.e. May (spring) or August (summer) 2015, from the total fungal community (TOT) at the Molln site, determined by random forest modelling

**OTU**	**Phylum**	**Genus**	**Species**	**Guild[a]**	**May 2015[b]**	**Aug 2015[c]**	**Diff.[d]**	**Import.[e]**
OTU_189	Ascomycota	*Penicillium*	*swiecickii*	SAP	0.009	0.122	0.11	19.5
OTU_29	Ascomycota	*Neonectria*	*coccinea/faginata/punicea*	PAT	0.003	0.184	0.18	15.7
OTU_130	Mortierellomycota	*Mortierella*	sp.	SAP	0.033	0.236	0.20	15.5
OTU_46	Mortierellomycota	*Mortierella*	*aff. sclerotiella*	SAP	0.182	0.843	0.66	13.7
OTU_327	Chytridiomycota	Rhizophydiales		NA	0.004	0.098	0.09	11.0
OTU_30	Basidiomycota	*Clavulina*	*cristata*	SYM	0.563	0.655	0.09	10.9
OTU_118	Mortierellomycota	*Mortierella*	sp.	SAP	0.099	0.402	0.30	10.6
OTU_66	Basidiomycota	*Hygrophorus*	*discoxanthus*	SYM	0.599	0.028	-0.57	10.6
OTU_710	Chytridiomycota	*Operculomyces*	sp.	SAP	0.001	0.055	0.05	9.9
OTU_400	Chytridiomycota	Rhizophydiales		NA	0.002	0.049	0.05	9.0
OTU_489	Chytridiomycota	Rhizophydiales		NA	0.007	0.098	0.09	9.0

[a] Ecological Guild: SAP, saprotrophic; SYM, symbiotic; PAT, potentially plant pathogenic; NA, not assigned; [b] Average relative abundance (%) in May 2015; [c] Average relative abundance (%) in August 2015; [d] Difference in average relative abundance; [e] Statistical significance of difference (uncorrected t-test); [e] Importance in random forest modelling – Mean Decrease in Accuracy

S4 Most important OTUs for the prediction of sampling date, i.e. May or August 2015, from the symbiotic fungal community (SYM) at the Molln site, determined by random forest modelling

				May 2015		Aug 2015			
**OTU**	**Phylum**	**Genus**	**Species**	**Mean[a]**	**Disp.[b]**	**Mean**	**Disp.**	**Diff.[c]**	**Import.[d]**
OTU_30	Basidiomycota	*Clavulina*	*cristata*	3.13	42.26	2.72	33.76	-0.41	24.2
OTU_7	Basidiomycota	*Inocybe*	*maculata*	9.60	58.15	6.37	20.08	-3.23	19.1
OTU_66	Basidiomycota	*Hygrophorus*	*discoxanthus*	3.39	23.82	0.22	0.81	-3.18	17.0
OTU_45	Basidiomycota	Sebacinaceae		0.15	3.74	3.33	46.24	3.19	11.8
OTU_269	Basidiomycota	*Tomentella*	*pilosa*	1.12	16.74	0.05	0.76	-1.07	11.2
OTU_11	Ascomycota	Hyaloscyphaceae		14.16	16.25	8.05	7.68	-6.10	11.1
OTU_17	Basidiomycota	*Suillellus*	*luridus*	8.39	69.56	3.97	41.01	-4.43	10.7
OTU_27	Ascomycota	Hyaloscyphaceae		7.10	7.44	4.03	3.45	-3.07	10.4
OTU_56	Basidiomycota	*Hymenogaster*	*citrinus*	0.26	1.31	2.47	19.37	2.21	10.2
OTU_113	Basidiomycota	*Inocybe*	*splendens*	0.29	3.80	1.79	36.29	1.50	7.6
OTU_44	Basidiomycota	Sebacinaceae		0.97	2.39	4.49	18.66	3.52	6.3

[a] Mean relative abundance (%); [b] Dispersion in SYM subset; [c] Difference in mean relative abundance; [d] Statistical significance of difference (uncorrected t-test); [d] Importance in random forest modelling – Mean Decrease in Accuracy

Table S5 OTUs responding to changes in soil pH and/or soil organic carbon content (% C_{org}) at the Molln site resulting in <7.5% mean decrease in accuracy as determined by random forest modelling

					pH		C_{org}	
**OTU**	**Phylum**	**Genus**	**Species**	**Guild**[a]	**ρ**[b]	**Imp.**[c]	**ρ**	**Imp.**
OTU_55	Mortierellomycota	*Mortierella*	*pseudozygospora*	SAP	**-0.546**	7.9	**-0.585**	20.0
OTU_47	Mortierellomycota	*Mortierella*	aff. *elongata*	SAP	**-0.578**	19.8	**-0.531**	7.0
OTU_320	Mortierellomycota	*Mortierella*	sp.	SAP	**0.535**	1.5	**0.613**	17.6
OTU_20	Ascomycota	Leotiomycetes		NA	**0.673**	13.5	**0.596**	15.3
OTU_83	Ascomycota	*Meliniomyces*	sp.	SAP	**-0.658**	14.8	-0.288	2.6
OTU_76	Ascomycota	*Pleotrichocladium*	*opacum*	PAT	**-0.431**	12.4	**-0.453**	4.7
OTU_230	Ascomycota	*Tetracladium*	aff. *setigerum*	SAP	0.011	0.4	**-0.340**	11.9
OTU_135	Mortierellomycota	*Mortierella*	sp.	SAP	**-0.626**	9.8	**-0.574**	11.4
OTU_57	Ascomycota	Hyaloscyphaceae		SYM	**-0.592**	10.9	**-0.363**	2.5
OTU_128	Ascomycota	Helotiales		NA	**-0.590**	10.7	**-0.401**	2.7
OTU_476	Basidiomycota	*Phyllozyma*	sp.	SAP	**-0.371**	10.7	**-0.368**	-0.7
OTU_118	Mortierellomycota	*Mortierella*	sp.	SAP	0.163	1.4	0.081	10.4
OTU_6	Basidiomycota	*Saitozyma*	*podzolica*	SAP	**0.440**	9.4	**0.489**	10.0
OTU_303	Ascomycota	Leotiomycetes		NA	**0.413**	0.2	**0.557**	9.9
OTU_526	Ascomycota	Xylariales		SAP	**-0.649**	9.8	**-0.486**	4.0
OTU_838	Fungi i.s.	Fungus		NA	**-0.540**	9.6	-0.250	-1.5
OTU_3	Ascomycota	Leotiomycetes		NA	**0.605**	9.3	**0.548**	9.6
OTU_179	Chytridiomycota	*Rhizophydium*	sp.	SAP	-0.307	5.9	**-0.514**	8.6
OTU_18	Ascomycota	Hyaloscyphaceae		SAP	0.051	1.4	**-0.379**	8.5
OTU_10	Ascomycota	*Exophiala*	sp.	SAP	-0.256	1.0	**-0.333**	8.4
OTU_379	Chytridiomycota	Rhizophydiales		NA	-0.063	0.1	**-0.385**	8.0
OTU_35	Ascomycota	Leotiomycetes		NA	-0.238	1.6	**-0.482**	7.9
OTU_1400	Mortierellomycota	*Mortierella*	aff. *exigua*	SAP	**-0.502**	4.2	**-0.563**	7.8
OTU_1096	Ascomycota	*Tetracladium*	aff. *setigerum*	SAP	**-0.402**	5.3	**-0.506**	7.6
OTU_121	Ascomycota	Helotiales		NA	0.108	0.4	**0.487**	7.5

[a] Ecological Guild: SAP, saprotrophic; SYM, symbiotic; PAT, potentially plant pathogenic; NA, not assigned; [b] Spearman's Rank Correlation Coefficient ρ for correlation between environmental variables pH or % $C_{org.}$ and relative OTU abundance, highly significant values at $p < 0.001$ are highlighted in bold; [c] Importance in random forest modelling (Mean Decrease in Accuracy), only OTUs with Importance ≥ 7.5 for pH or C_{org} are shown.

3 Supplementary Figures

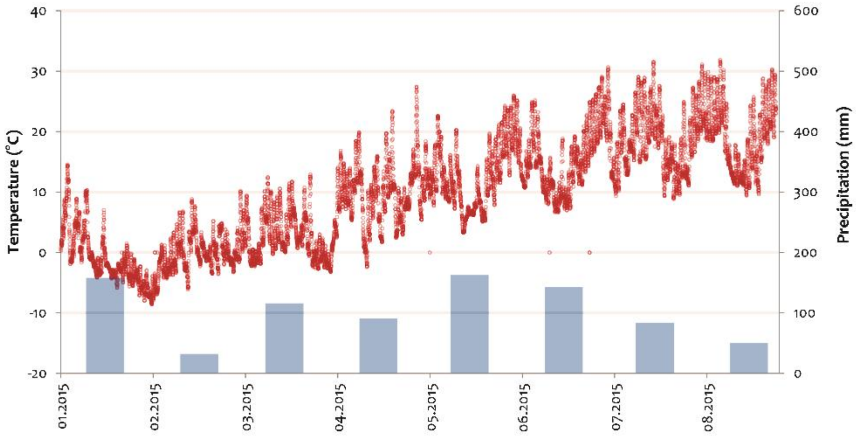

Figure S1 Air temperature (°C; dots) and precipitation (mm; bars) from January to August 2015 from a weather station in Molln, Upper Austria; see Figure S5b for gravimetric soil moisture at sampling dates

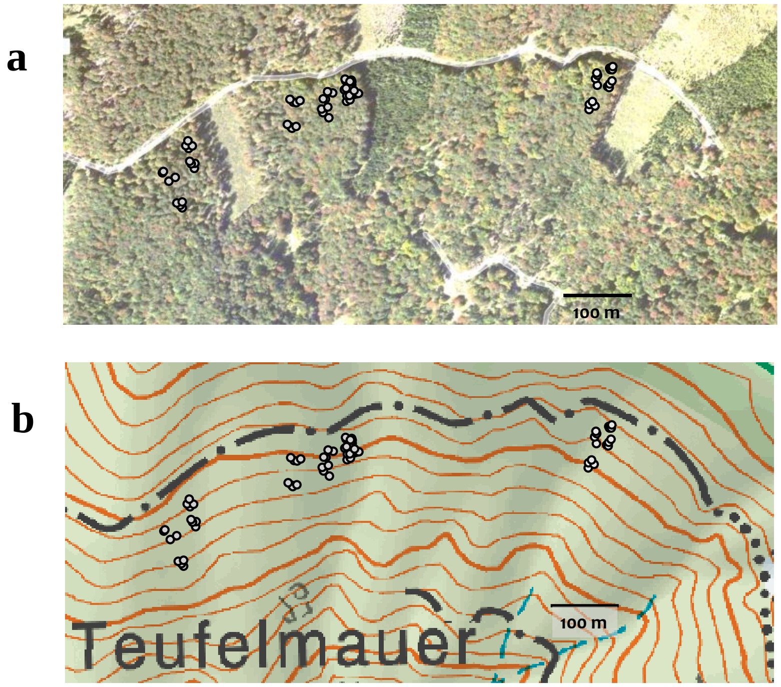

Figure S2 Experimental site in Molln, Upper Austria (47°49'08" N, 14°23'34" E). Position of 64 subplots on a satellite image (**a**; Google Maps) and a topographic map (**b**; www.austrianmap.at)

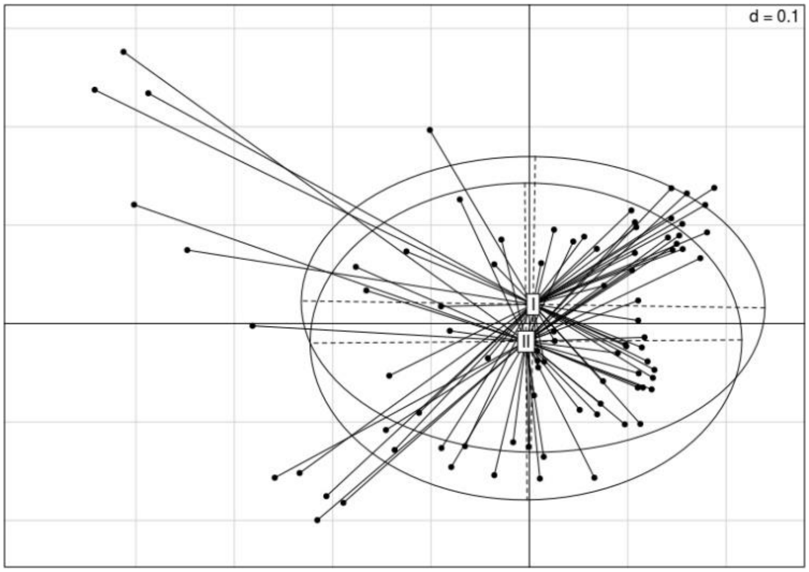

Figure S3 PCoA of the GUniFrac distances with annotated extraction factors (I and II). Samples from May 2015 were extracted twice and analysed separately for fungal community composition

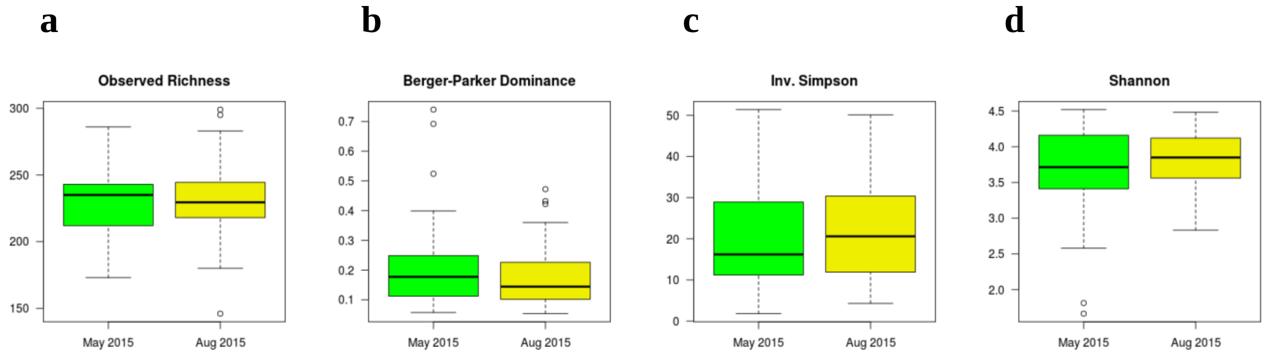

Figure S4 Indicators of fungal diversity at the Molln site in May (spring) and August (summer) 2015**,** i.e. observed richness **(a)**, Berger-Parker Dominance Index (relative abundance of the most abundant OTU) **(b)**, Simpson's Inverse Diversity Index **(c)**, and Shannon Diversity Index **(d).** Boxplots were calculated from single soil samples (May: n = 48; August: n = 64), outliers are marked as circles

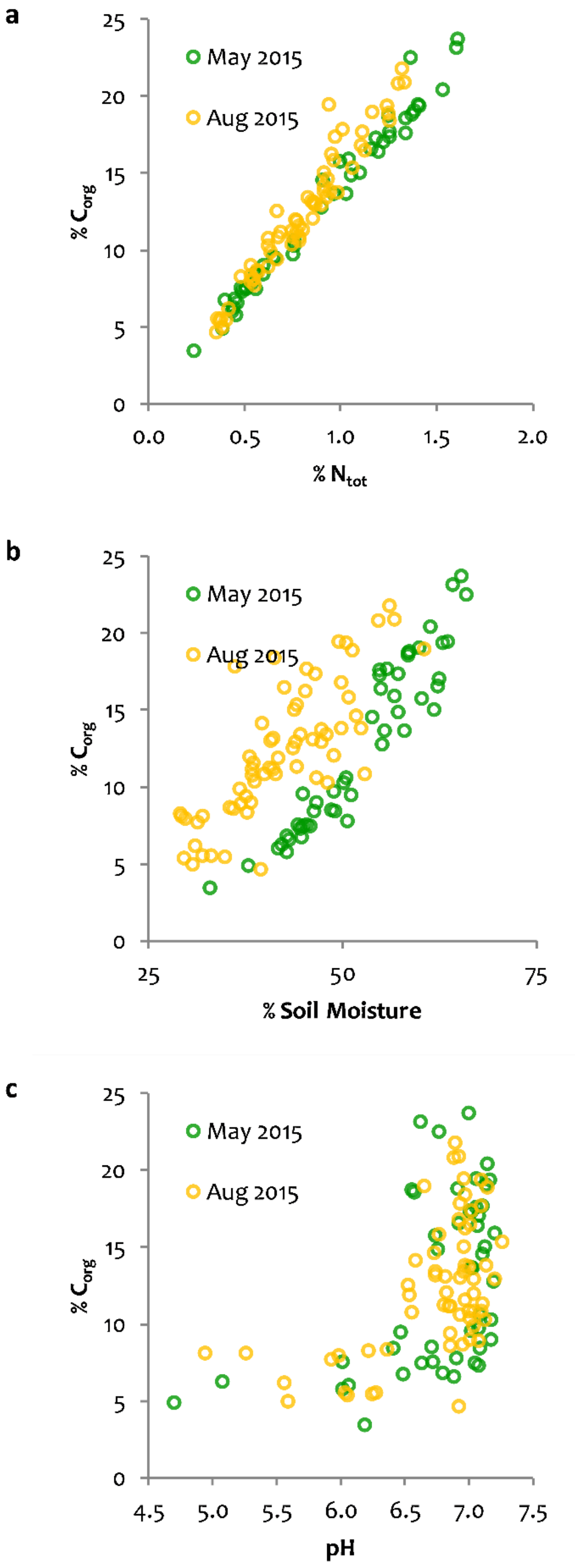

Figure S5 Correlations between organic carbon content (% C_{org}) and total nitrogen content (% N_{tot}) **(a)**, % soil moisture **(b)** and pH($CaCl_2$) **(c)** in soil samples of the Molln site from May (spring) and August (summer) 2015

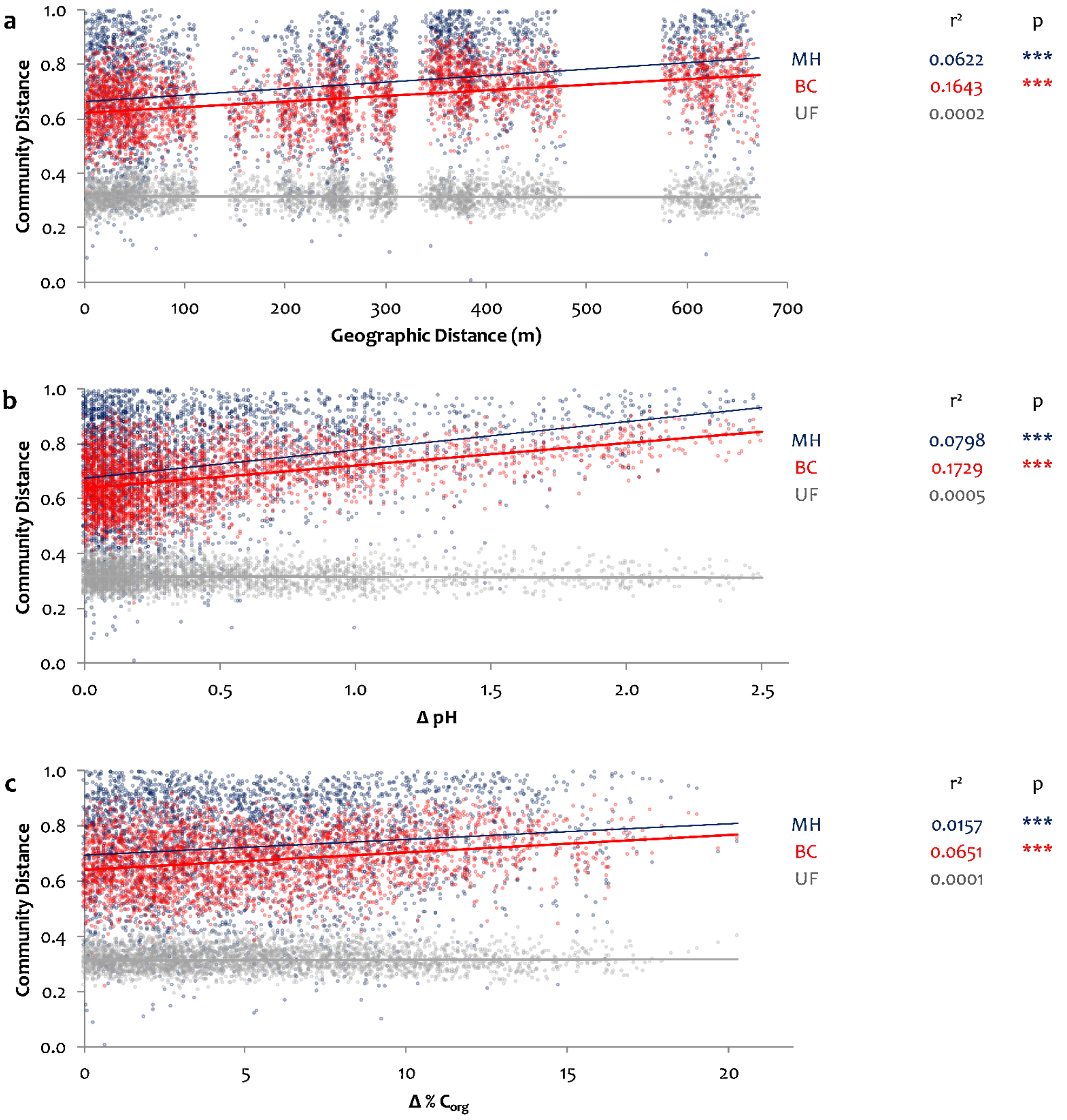

Figure S6 Environmental influences on β-diversity at the Molln site. Three different indices of β-diversity – Morisita-Horn (MH, blue), Bray-Curtis (BC, red) and UniFrac (UF, grey) – were calculated from the fungal community data and plotted against the geographic distance **(a)**, and the differences in pH($CaCl_2$) **(b)**, and organic soil carbon (% C_{org}) **(c)**. Regression coefficients (r^2) and significances are given in the corresponding colors next to the subpanel. All subpanels contain data from both seasons (May, August), where β-diversity indices were only calculated within the season

4 Supplementary References

1. Kobler J, Jandl R, Dirnböck T, Mirtl M, Schindlbacher A (2015) Effects of stand patchiness due to windthrow and bark beetle abatement measures on soil CO_2 efflux and net ecosystem productivity of a managed temperate mountain forest. Eur J Forest Res 134 (14):683-692. doi:10.1007/s10342-015-0882-2

2. ÖNORM L 1080 (1999) Chemische Bodenuntersuchungen – Bestimmung des organischen Kohlenstoffs durch trockene Verbrennung. Austrian Standards Institute

3. ÖNORM L 1084 (1999) Chemische Bodenuntersuchungen – Bestimmung von Carbonat. Austrian Standards Institute

4. IUSS Working Group WRB (2006) World reference base for soil resources 2006, vol 2nd edition. World Soil Resources Reports No. 103. . FAO Rome

5. Zanella A, Jabiol B, Ponge JF, Sartori G, De Waal R, Van Delft B, Graefe U, Cools N, Katzensteiner K, Hager H, Englisch M (2011) A European morpho-functional classification of humus forms. Geoderma 164 (3–4):138-145. doi:10.1016/j.geoderma.2011.05.016

6. Böhm W (1979) Profile Wall Methods. In: Böhm W (ed) Methods of Studying Root Systems. Springer, Berlin, Heidelberg. doi:10.1007/978-3-642-67282-8_6

7. ÖNORM L 1085 (2013) Chemische Bodenuntersuchungen - Verfahren zur Extraktion von Elementen mit Salpetersäure-Perchlorsäure-Gemisch. Austrian Standards Institute

8. Feinstein LM, Sul WJ, Blackwood CB (2009) Assessment of bias associated with incomplete extraction of microbial DNA from soil. Appl Environ Microbiol 75 (16):5428-5433. doi:10.1128/AEM.00120-09

9. Chen J (2018) Package 'GUniFrac'. https://cranr-projectorg/web/packages/GUniFrac/GUniFracpdf

10. Oksanen J, Blanchet FG, Friendly M, Kindt R, Legendre P, McGlinn D, Minchin PR, O'Hara RB, Simpson GL, Solymos P, Stevens MHH, Szoecs E, Wagner H (2018) Package "vegan". R Packag ver 20–8. https://cranr-projectorg/web/packages/vegan/veganpdf

11. Tedersoo L, Bahram M, Põlme S, Kõljalg U, Yorou NS, Wijesundera R, Villarreal Ruiz L, Vasco-Palacios AM, Thu PQ, Suija A, Smith ME, Sharp C, Saluveer E, Saitta A, Rosas M, Riit T, Ratkowsky D, Pritsch K, Põldmaa K, Piepenbring M, Phosri C, Peterson M, Parts K, Pärtel K, Otsing E, Nouhra E, Njouonkou AL, Nilsson RH, Morgado LN, Mayor J, May TW, Majuakim L, Lodge DJ, Lee SS, Larsson KH, Kohout P, Hosaka K, Hiiesalu I, Henkel TW, Harend H, Guo LD, Greslebin A, Grelet G, Geml J, Gates G, Dunstan W, Dunk C, Drenkhan R, Dearnaley J, De Kesel A, Dang T, Chen X, Buegger F, Brearley FQ, Bonito G, Anslan S, Abell S, Abarenkov K (2014) Fungal biogeography. Global diversity and geography of soil fungi. Science 346 (6213):1256688. doi:10.1126/science.1256688

12. White T, Bruns T, Lee S, Taylor J (1990) Amplification and direct sequencing of fungal ribosomal RNA genes for phylogenetics. In: Innis M, Gelfand D, Sninsky J, White T (eds) PCR protocols: a guide to methods and applications. Academic Press, United States, pp 315-322

13. Keiblinger KM, Schneider M, Gorfer M, Paumann M, Deltedesco E, Berger H, Jochlinger L, Mentler A, Zechmeister-Boltenstern S, Soja G, Zehetner F (2018) Assessment of Cu applications in two contrasting soils-effects on soil microbial activity and the fungal community structure. Ecotoxicology 27 (2):217-233. doi:10.1007/s10646-017-1888-y

14. Tedersoo L, Anslan S, Bahram M, Põlme S, Riit T, Liiv I, Kõljalg U, Kisand V, Nilsson H, Hildebrand F, Bork P, Abarenkov K (2015) Shotgun metagenomes and multiple primer pair-barcode combinations of amplicons reveal biases in metabarcoding analyses of fungi. MycoKeys 10. doi:10.3897/mycokeys.10.4852

15. Kraus D (2014) Consolidated data analysis and presentation using an open-source add-in for the Microsoft Excel® spreadsheet software. Medical Writing 23 (1):25-28. doi:10.1179/2047480613Z.000000000181

16. Goldmann K, Schroter K, Pena R, Schoning I, Schrumpf M, Buscot F, Polle A, Wubet T (2016) Divergent habitat filtering of root and soil fungal communities in temperate beech forests. Sci Rep 6:31439. doi:10.1038/srep31439

17. Tedersoo L, Smith ME (2013) Lineages of ectomycorrhizal fungi revisited: Foraging strategies and novel lineages revealed by sequences from belowground. Fungal Biol Rev 27 (3):83-99. doi:10.1016/j.fbr.2013.09.001
